# Evaluation of ToF-SIMS imaging for semi-quantitative mapping of BODIPY-labeled fibronectin surface gradients

**DOI:** 10.1039/d5an00962f

**Published:** 2026-01-19

**Authors:** Chao Liu, Tae Kyong John Kim, Douglas H. Wu, Radhika Atit, Rodrigo A. Somoza, Samuel E. Senyo

**Affiliations:** a Department of Biomedical Engineering, Case Western Reserve University Cleveland OH USA ssenyo@case.edu; b Swagelok Center for Surface Analysis of Materials, Case Western Reserve University Cleveland OH USA; c Medical Scientist Training Program, Case Western Reserve University Cleveland OH USA; d Department of Biology, Case Western Reserve University Cleveland OH USA; e Department of Biology, Skeletal Research Center, Case Western Reserve University Cleveland OH USA; f Center for Modular Manufacturing of Structural Tissues, Case Western Reserve University Cleveland OH USA

## Abstract

Microfluidic platforms offer controlled microenvironments for studying cell migration such as haptotaxis. In many gradient-based assays, protein gradients are first visualized using higher concentrations of fluorescent labels, since gradients formed at biologically relevant ligand densities often fall below the detection limits of conventional imaging methods. In this study, we demonstrate the feasibility of characterizing fibronectin gradients using a more sensitive, high-resolution approach with Time-of-Flight Secondary Ion Mass Spectrometry (ToF-SIMS). Our methods increase analytical sensitivity to fibronectin gradients formed on commonly used synthetic surfaces to better elucidate physiological mechanisms and ensure experimental reproducibility. We utilized a microfluidic chip with a silicone housing placed on an optically clear plastic microscope slide designed for live-cell microscopy. Slides were coated with fibronectin incorporating surrogate labels for ToF-SIMS analysis to investigate spatial distribution. To enhance signal detection, fibronectin was labeled or conjugated with copper, bromine, and fluorine-containing BODIPY as surrogate elements. Among the tested labels, BODIPY-fibronectin (BODIPY-FN) provided the lowest background signal, enabling fluorescence-based detection at concentrations of 10 µg mL^−1^ or higher, whereas ToF-SIMS demonstrated greater sensitivity, detecting fibronectin gradients at concentrations of 1 µg mL^−1^ or higher. Semi-quantitative measurements using imaging mass spectrometry confirmed a graded distribution of fibronectin within the physiologically relevant “haptotaxis zone”, with detection sensitivity exceeding the limits of standard fluorescent microscopy. These results establish ToF-SIMS as a valid method for detecting surrogate-labeled ligand gradients, providing a reliable approach for future quantitative ligand measurements.

## Abstract 2

A novel semi-quantitative imaging mass spectrometry approach using ToF-SIMS enables high-resolution, sensitive mapping of fibronectin gradients in microfluidic haptotaxis assays beyond the limits of fluorescence microscopy.

## Graphical abstract

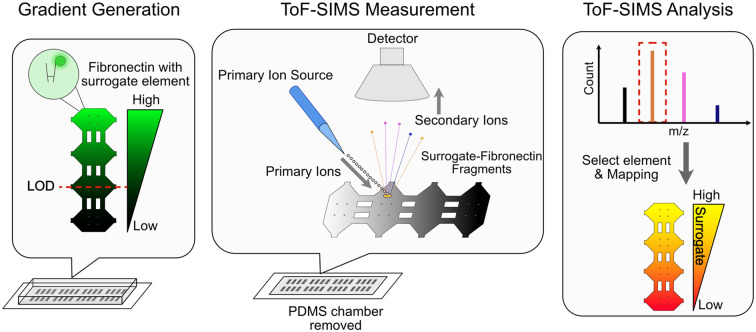

## 1. Introduction

Cell migration in response to extracellular matrix (ECM) cues is fundamental to many biological processes including tissue development, wound repair and disease progression.^1,2^ Haptotaxis is a key mechanism of migration involving directed movement of cells along gradients of substrate-bounded ECM ligands such as fibronectin (FN). Haptotaxis cues offer stable, spatially localized guidance that regulates cell adhesion, polarization, and motility in contrast to transient soluble signals with chemotaxis.^3–5^ Reliable characterization of gradient cues is essential for evaluating cell–substrate interactions, which in turn inform the rational design of interactive biomaterials. While numerous studies have demonstrated haptotaxis in microfluidic chips, a common limitation is their reliance on high concentrations of fluorescent labeling to visualize gradients. These techniques often fail to recapitulate gradients at lower, physiologically relevant ligand densities at which cells exhibit directed migration.^4,6–8^ There is a need to investigate alternative analytical approaches that offer higher precision and greater sensitivity with lower detection limits.

Time-of-Flight Secondary Ion Mass Spectrometry (ToF-SIMS) is a highly sensitive surface analysis technique capable of generating high-resolution chemical images and detailed spatial distributions of molecular species on a substrate.^9^ Its capacity to detect a broad range of surface-bound compounds makes it an attractive tool for investigating ligand gradients. However, direct analysis of complex proteins such as fibronectin remains a significant challenge for biological applications.^10^ This is due to its large molecular size, flexibility, extensive fragmentation under ion bombardment, and the absence of a unique, identifiable peak in the ToF-SIMS spectrum. Moreover, background interference from materials such as polydimethylsiloxane (PDMS), which is commonly used in the fabrication of microfluidic chips, further complicates spectral interpretation and obscures relevant molecular signals.^11–14^ Collectively, these factors pose significant obstacles to the direct detection of fibronectin ligand gradients using ToF-SIMS. To address low signal-to-noise ratios, multivariate techniques such as principal component analysis (PCA) are often employed to extract meaningful spectral information. In addition, complementary elemental labeling methods that generate distinct, identifiable peaks can further improve the reliability of mass spectrometry imaging in life sciences.^9,10,12,15^

In ToF-SIMS, surrogate labeling with elements such as bromine (Br), fluorine (F), and copper (Cu) and stable isotopes enhances molecular detection and spatial resolution in complex biological systems. These elemental labels produce distinct mass signals and are rare in biological backgrounds, enabling precise localization of labeled compounds. For example, brominated furanones have been used as labels to assess the uniformity of antibacterial furanone grafting on surfaces, leveraging unique isotopic pattern of bromine for clear identification.^16^ Similarly, fluorinated nanobodies have been developed for targeted imaging of cellular proteins, allowing high-resolution visualization using ^19^F-specific signals.^17^ Additionally, stable isotope labeling, such as ^15^N incorporation, enables tracking of cellular processes like protein synthesis and cell division, offering a powerful approach to study dynamic biological systems.^18,19^ These labeling strategies significantly improve the analytical power of ToF-SIMS in biological research.

In this study, we explored labeling strategies to enhance the detection of fibronectin (FN) using ToF-SIMS. FN was conjugated with three different labels enriched in distinct elements: bicinchoninic acid (BCA) providing copper (Cu), eosin supplying bromine (Br), and BODIPY introducing fluorine (F). We assessed the detection limits of each labeling method and compared them with conventional fluorescence-based detection. Among the tested labels, BODIPY-conjugated FN facilitated the highest detection sensitivity, producing distinct signals in both mass spectra and spatial distribution maps. To evaluate this approach in a biologically relevant context, we used a custom-designed microfluidic chip capable of generating FN gradients on optically transparent, cell-compatible plastic substrates suitable for on-chip live-cell imaging. After gradient formation *via* diffusion, the PDMS chamber was detached from the plastic slide, and the BODIPY-FN gradient was evaluated using a semi-quantitative method of ToF-SIMS, determining both the spatial distribution and surface density. In summary, ToF-SIMS enables highly sensitive and high-resolution measurement of element-tagged fibronectin gradients on surfaces. This sensitive labeling and detection strategy provides a robust platform for future studies involving gradient generation and surface-bound protein quantification.

## 2. Materials and methods

### 2.1. Microfluidic chip fabrication

The microfluidic chips were fabricated using a two-layer soft lithography process, as previously described.^4,20^ To produce the top control layer chip, PDMS and curing agent (Ellsworth Adhesives 2085925), were mixed at a 5 : 1 (base : crosslinker) ratio, degassed, and baked at 80 °C for 30 minutes. Control layer inlets were punched using a 1 mm biopsy punch on a custom punching station. For the flow layer, PDMS and curing agent were mixed at a 20 : 1 ratio, degassed, spin-coated onto the flow layer master mold at 1000 rpm for 60 seconds, allowed to reflow for 20 minutes, and partially cured at 80 °C for 6 minutes. The control layer was carefully aligned and bonded to the flow layer under a stereo-microscope, followed by baking for 30 minutes. The assembled chip was then removed from the mold, and flow layer inlets were punched. The chip was treated with oxygen plasma (20 W, 30 s), and bonded to plastic slides (Ibidi 10812) that were pre-treated with oxygen plasma (30 W, 30 s) followed by a 20-minute incubation in 1% (v/v) (3-aminopropyl)triethoxysilane (APTES). The chip was then post-baked for 1 hour at 80 °C. Finally, the chip was stored at room temperature for 2 weeks for material stabilization to prevent permanent valve collapse during operation.

### 2.2. FITC-dextran intensity measurement

FITC-dextran (Sigma, F2733) was diluted in 1× PBS to final concentrations of 1, 10, 100, and 1000 μg mL^−1^ and loaded into a microfluidic chip. Each concentration was tested in quadruplicate, along with a 1× PBS control to account for background fluorescence. Fluorescence intensity was measured by manually defining regions of interest (ROIs), and average intensities were quantified using FIJI/ImageJ (version 2.14.0/1.54f). The mean fluorescence intensity at each concentration, along with the background control, was plotted using a custom script in RStudio (version 4.3.1). A dotted reference line was drawn between the average intensities of the 100 and 1000 μg mL^−1^ samples to indicate the projected trend for lower concentrations.

### 2.3. BCA staining for surface fibronectin labeling

Plastic slides (Ibidi 10812) underwent the same surface treatment described previously, including oxygen plasma treatment followed by incubation in 1% (v/v) APTES. Treated slides were cut into smaller pieces (1 cm × 1 cm) and coated overnight with fibronectin solutions at concentrations of 1, 10, 50, and 100 μg mL^−1^ (diluted in deionized water), alongside a water-only control. To enable subsequent detection of fibronectin by ToF-SIMS, the bicinchoninic acid (BCA) assay reagent (Thermo Scientific, 23225) was used as a surrogate label containing copper. Following the manufacturer's protocol, a 50 : 1 mixture of Reagent A to Reagent B was prepared and applied to the fibronectin-coated and control surfaces, which were incubated at 37 °C for 30 minutes to allow reduction of Cu^2+^ to Cu^+^ by the adsorbed protein. After incubation, the slides were thoroughly rinsed with deionized (DI) water and analyzed by ToF-SIMS to detect copper as an indirect marker of fibronectin adsorption.

### 2.4. Eosin staining for surface fibronectin labeling

Plastic slides were treated and cut into small pieces (1 cm × 1 cm), then coated overnight with fibronectin at varying concentrations (1, 10, 50, and 100 µg mL^−1^), along with a water-only control, as described in the previous methods section. Eosin Y dye (StatLab, SL402) was applied to the surfaces for 1 minute, followed by two washes with 95% ethanol and two additional washes with 100% ethanol to remove any residual dye. Eosin Y binds to basic amino acids in proteins and contains bromine (Br), which serves as a surrogate marker for fibronectin detection in subsequent ToF-SIMS analysis.

### 2.5. BODIPY conjugation to fibronectin

Fibronectin (Sigma, FC010; 1 mL, 1 mg mL^−1^ in TBS) was concentrated to 5 mg mL^−1^ using a 10 kDa MWCO Pierce protein concentrator with 2–3 centrifugation cycles at 12 000*g* for 5 minutes each. To remove reactive amines from the buffer, a two-step buffer exchange into 0.1 M bicarbonate (pH 8.5) was performed using Zeba Spin desalting columns (ThermoFisher, 89877; 7 kDa MWCO). Columns were first equilibrated by flushing with bicarbonate buffer three times at 1500*g* for 1 minute, after which the fibronectin solution was applied and centrifuged at 1500*g* for 2 minutes. This process was repeated using a second set of freshly prepared columns to ensure >99% amine removal. The desalted fibronectin was then mixed with 10 µL of BODIPY-FL STP ester (ThermoFisher, B10006; 10 mg mL^−1^ in DI water), protected from light, and incubated for 1 hour on an orbital shaker at room temperature. Excess dye and buffer salts were removed through two additional passes through Zeba desalting columns, continuing until the column bed no longer retained visible yellow-green coloration (Fig. 2A). Final purification was achieved by concentrating the conjugate to approximately 100 µL using a protein concentrator, followed by repeated washes and resuspension in DI water. The conjugated protein appeared as an orange solution. To determine precise concentration, the sample was transferred to a pre-weighed tube, flash-frozen in liquid nitrogen, lyophilized for 24 hours, and the dry mass was recorded for concentration calculation (Fig. 2B).

### 2.6. Determination of BODIPY-FN degree of labeling

The BODIPY-fibronectin conjugate, prepared at its working concentration in DI water, was analyzed using an Implen Nanophotometer NP80. A 1 µL aliquot of each undiluted sample was placed on the quartz window, and UV-Vis spectra were recorded from 200–900 nm with baseline correction at 650 nm (Fig. 3). A virtual 15× dilution was applied, and absorbance values were normalized to a 1 cm path length.

The degree of labeling (DOL) was calculated from *A*_280_ and the fluorophore absorbance maximum (*A*_max_) at 504 nm using eqn (1). Extinction coefficients for protein (*ε* = 594 000 M^−1^ cm^−1^) and dye (*ε*′ = 79 700 M^−1^ cm^−1^) and a correction factor for dye absorbance at 280 nm (CF = 0.04) were applied according to manufacturer specifications.1
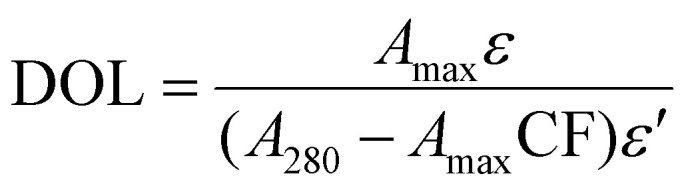


The known protein sequence of fibronectin contains 78 lysine residues per subunit (∼156 per dimer),^21^ which was used to estimate the percentage of reactive amines labeled.

### 2.7. BODIPY-FN fluorescent intensity measurement

BODIPY-conjugated fibronectin (BODIPY-FN) was serially diluted in DI water to final concentrations of 500, 100, 10, and 1 μg mL^−1^, along with a DI water control. Each condition was incubated in a 96-well plate (three replicates per condition) for 3 hours at room temperature. Following incubation, wells were washed with 1× PBS. Fluorescence was measured in endpoint mode using a Synergy H1 plate reader (excitation: 485 nm; emission: 528 nm). The mean of the fluorescence intensities was plotted against BODIPY-FN concentrations using MATLAB (R2022b).

### 2.8. Fibronectin gradient generation and fluorescence intensity analysis

BODIPY-FN gradients were generated in microfluidic chips using a diffusion-based approach, as previously described.^4^ BODIPY-FN was diluted to final concentrations of 100 and 500 μg mL^−1^ and perfused into the source chamber (chamber D) of the microfluidic device. To maintain a stable source concentration, the BODIPY-FN solution was repeatedly perfused into the closed source chamber, and the valves between the source chamber and the remaining chambers were periodically opened every 30–40 minutes to enable fibronectin diffusion over a 24-hour period. After gradient formation, all chambers were thoroughly washed with DI water before ToF-SIMS analysis. The resulting gradient was visualized using fluorescence microscopy (Olympus IX81 microscope with a motorized stage), and fluorescence intensity profiles were quantified by drawing three lines of regions of interest (ROIs) spanning the four chambers (Fig. S5A). Average line profiles were generated by averaging data from four technical replicates using a custom MATLAB script (Fig. S5B), as described previously.^4^

### 2.9. BODIPY-FN standard concentration ToF-SIMS measurement

Plastic slides were treated with oxygen plasma followed by incubation in 1% APTES solution, then cut into smaller pieces (1 cm × 1 cm) as described in the previous methods section. BODIPY-FN was diluted in DI water to concentrations of 0.1, 1, 10, 25, 100, and 500 μg mL^−1^, with a DI water-only control included. These solutions were applied to the prepared plastic slides and incubated overnight, followed by a rinse with DI water to remove unbound material. The coated slides were subsequently analyzed by ToF-SIMS, using the 19[F]^−^ ion signal as a surrogate marker for BODIPY-FN (fibronectin) detection.

### 2.10. ToF-SIMS measurement

ToF-SIMS analyses were performed using a TRIFT V nanoTOF instrument (Physical Electronics). Samples were loaded into the fast entry chamber, which was evacuated using a turbomolecular pump to pressures below 5 × 10^−6^ Torr before transferring them into the analytical chamber. The analytical chamber achieved a base pressure of approximately 2 × 10^−9^ Torr using a combination of turbomolecular and ion pumps. A 30 keV Ga^+^ pulsed primary ion source was used for all measurements. Both bunched mode (mass resolution mode) and unbunched mode (spatial resolution mode) were used to acquire spectral data. Standards for Cu and Br were measured in bunched mode to ensure high mass resolution, while F was measured in unbunched mode for both the BODIPY-FN standard and gradient to preserve spatial resolution.

For standard samples, spectral data were acquired by scanning 200 µm × 200 µm regions. Within each scanned region, four regions of interest (ROIs), each approximately 100 µm × 100 µm, were defined to quantify total ion counts and surrogate ion signals. For chip samples, mosaic mapping was performed with a step size of 0.2 mm to capture the complete area of the gradient encompassing four chamber structures, while a finer step size of 0.1 mm was utilized to achieve higher-resolution imaging of the two chambers with lower protein concentrations. All measured surrogate ion counts were normalized to the corresponding total ion counts for each ROI. Acquisition times were carefully controlled to remain within the static SIMS limit (total ion dose <1012 ions per cm^2^). Mass spectra were collected in the mass-to-charge (*m*/*z*) range of 0–1850, using positive polarity for BCA-labeled samples and negative polarity for Eosin- and BODIPY-labeled samples. Charge compensation during analysis was achieved using 10 eV electrons. Calibration for positive-ion mode was performed using H, Na, and K peaks, while CH, O, OH, and C2H peaks were used for calibration in negative-ion mode. All data analyses were conducted using WinCadenceN 1.8.1 software (Physical Electronics).

### 2.11. ToF-SIMS standard analysis

Sample data files from each staining strategy (Eosin Y, BCA, or BODIPY-FN) were loaded into the software and replayed to identify characteristic surrogate ion peaks corresponding to their surrogate elements: Br^−^ for Eosin Y, Cu^+^ for BCA, and F^−^ for BODIPY-FN. Spectral images illustrating surrogate element distributions were generated, and the heatmap intensity scales were uniformly adjusted across conditions to facilitate direct comparisons. Total ion counts and surrogate ion counts were measured within multiple ROIs for each condition, and normalized values (surrogate ion counts divided by corresponding total ion counts) were calculated. These data were summarized and presented as mean ± standard deviation in Tables S1–S3. Surrogate element peaks ([Cu]^+^ at *m*/*z* 62.9, [Br]^−^ at *m*/*z* 78.92, and [F]^−^ at *m*/*z* 18.998) were plotted as bar graphs using MATLAB (R2022b) with a consistent *y*-axis scale. Normalized surrogate element counts were further plotted as line graphs for comparative analysis.

### 2.12. ToF-SIMS gradient analysis

BODIPY-FN was used to generate a diffusion-based gradient within a microfluidic chip. After 24 hours of diffusion with either 100 or 500 μg mL^−1^ BODIPY-FN, the PDMS chamber was carefully removed, leaving only the plastic slide (Fig. S6A). The slide was then cut into smaller pieces for mounting onto the ToF-SIMS analysis stage (Fig. S6B and S6C). Mosaic mapping was performed on either the full four-chamber region or a higher-resolution two-chamber region. To analyze the spatial distribution of the fibronectin gradient, multiple rectangular ROIs were drawn in the direction of diffusion. Specifically, eight ROI regions were positioned at distinct locations along the *y*-axis and extended across the *x*-axis of the chambers. Within each ROI region, three rectangular ROIs were drawn to measure the F^−^ ion peak count and the total ion count. Normalized F^−^ ion counts from the three ROIs within each region were extracted, and the mean and standard deviation were calculated for each ROI region. These values were then plotted as a line graph to visualize the gradient profile.

### 2.13. Statistical analysis

Statistical analyses were performed using GraphPad Prism (version 8.0.1) and the results are presented in Fig. S3. For ToF-SIMS measurements of varying concentrations of BODIPY-FN, fibronectin labeled with surrogate BCA, fibronectin labeled with Eosin, and for fluorescence measurements of BODIPY-FN, group differences were assessed using one-way ANOVA followed by Dunnett's *post hoc* test, with comparisons made to the water control (0 µg mL^−1^). For BODIPY-FN gradient analysis, comparisons were made to ROI 1 region using one-way ANOVA with Dunnett's test. A *p*-value <0.05 was considered statistically significant.

## 3. Results and discussion

### 3.1. Results

#### 3.1.1. Limitations of fluorescence microscopy for visualizing fibronectin gradients

**Fig. 1:**
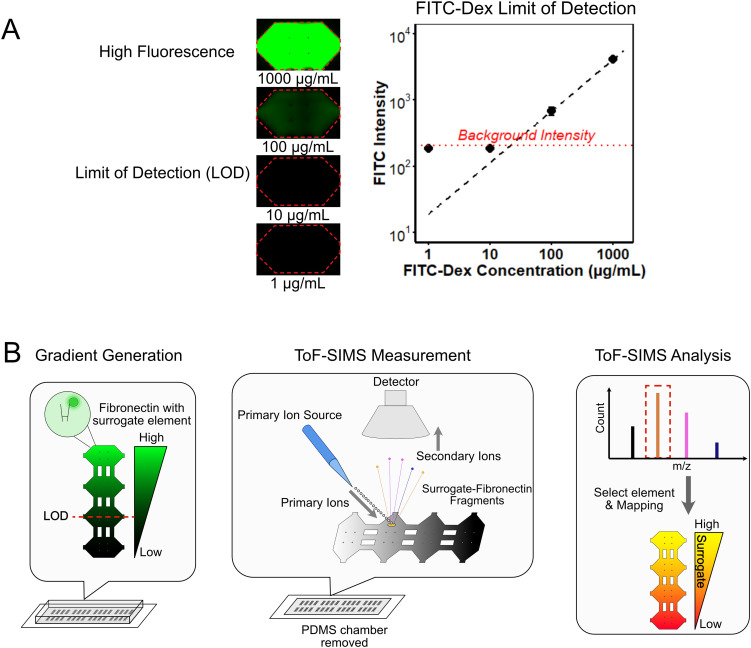
Schematic of ToF-SIMS measurement for a surrogate-labeled fibronectin gradient. (A) Fluorescent labeling using FITC-dextran shows high background signal, with a detection limit of approximately 10 µg ml^−1^ when compared to background intensity. (B) Fibronectin tagged with surrogate molecules enables detection through distinct elemental peaks in ToF-SIMS imaging, enhancing sensitivity and improving the visualization of the fibronectin gradient.

Fluorescent dyes are commonly used to visualize ligand gradients within microfluidic devices, enabling real-time assessment of diffusion dynamics and gradient formation. Fluorescein isothiocyanate (FITC), known for its high brightness and compatibility with biological assays, was used to establish the microscopy detection limit of FITC-dextran fluorescence. A standard curve was generated using known concentrations ranging from 1 to 1000 µg mL^−1^ in a microfluidic chip, previously developed for creating bound-ligand gradients to study haptotaxis.^4^ Fluorescence intensities were quantified and averaged across four technical replicates, then compared to background levels (Fig. 1A). FITC-dextran concentrations at or below 10 µg mL^−1^ were indistinguishable from background, defining the lower detection threshold. This finding is consistent with our previous observations that low concentrations of FITC-labeled or fluorescent antibody-probed fibronectin show poor visualization of the entire gradient field.^4^ Ideally, concentrations as low as 1–10 µg mL^−1^ should be detectable to confirm ligand gradient concentrations in the observed range where haptotaxis occurred in our studies.^4^ To overcome this limitation, we proposed ToF-SIMS as an alternative detection method. First, a surrogate molecule with specific ionizable elements would be conjugated to fibronectin, subsequently the conjugate would be used to establish a surface-bound ligand gradient within the microfluidic chip. Following gradient formation, the PDMS chamber would be removed, and the unobstructed sample slides then analyzed using ToF-SIMS. The ion peaks corresponding to the surrogate molecule could be normalized to the total ion signal, allowing for sensitive and spatially resolved mapping of the fibronectin gradient (Fig. 1B).

#### 3.1.2. Eosin and BCA labeling for ToF-SIMS-based fibronectin quantification

ToF-SIMS analysis has proven effective for characterizing extracellular matrix (ECM) proteins, including fibronectin. However, the resulting protein mass spectra are often complex, producing fragmented ion patterns that typically require multivariate methods such as principal component analysis (PCA) to distinguish specific protein types.^22,23^ In this study, we adopted a simplified and targeted strategy. We first evaluated two surrogate labeling approaches, bicinchoninic acid (BCA) and Eosin Y, to introduce distinct elemental signatures that could facilitate direct detection of fibronectin in ToF-SIMS spectra without relying on extensive spectral deconvolution. BCA was initially assessed because it forms a stable complex with protein-associated copper ions generated through reduction of Cu^2+^ by peptide residues. When fibronectin-coated slides were treated with BCA, ToF-SIMS detected a Cu^+^ signal at *m*/*z* 62.93, and normalized copper intensity increased progressively with fibronectin concentration (Fig. S1A, B, Fig. S3B and Table S1). However, appreciable copper signals were also detected in water controls (Fig. S1C), likely due to interactions between BCA and the substrate surface, and nearby organic fragments at *m*/*z* 62.99 and 63.03 interfered with accurate Cu^+^ quantification. These limitations reduced the specificity of copper as a surrogate signal for fibronectin. We next evaluated Eosin Y, an anionic dye that electrostatically binds to positively charged residues in fibronectin and contains four bromine atoms that generate a clear Br^−^ peak in negative ion mode. Br^−^ intensity increased with fibronectin concentration and exhibited minimal background interference (Fig. S2A–C, Fig. S3C and Table S2), demonstrating that Eosin Y can serve as a detectable small-molecule label. Nonetheless, its noncovalent binding and reduced sensitivity at lower fibronectin concentrations limited its ability to resolve shallow gradients. Together, these results indicated that although both surrogate dyes produced measurable signals, neither provided the sensitivity and specificity needed for accurate fibronectin gradient mapping, prompting the development of an alternative labeling strategy.

#### 3.1.3. BODIPY-fibronectin for fluorescence and ToF-SIMS quantification

To address this, we utilized BODIPY-conjugated fibronectin (BODIPY-FN), a probe compatible with both fluorescence imaging and mass spectrometry analysis. BODIPY-FL STP ester is a bright, photostable green fluorophore that covalently binds to primary amines, enabling high-resolution visualization of FN distribution. Notably, each BODIPY molecule contains six fluorine atoms, which are detectable at *m*/*z* 18.998 in negative ion mode by ToF-SIMS. In subsequent experiments, we evaluated whether the fluorine signal from BODIPY-FN could serve as a surrogate marker for FN, thereby enabling correlative analysis of its spatial distribution and surface concentration.

**Fig. 2:**
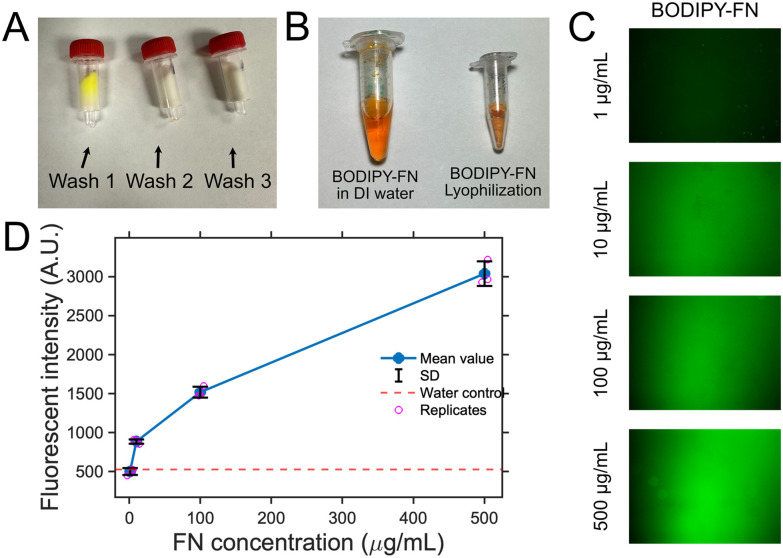
Fluorescence characterization of BODIPY-conjugated fibronectin (BODIPY-FN) across a range of concentrations. (A) Confirmation of complete removal of residual free BODIPY dye after conjugation, as indicated by the clear color of the desalting column following three successive washes. (B) Image of BODIPY-FN reconstituted in DI water and lyophilized into powder form. (C) Representative fluorescence images of BODIPY-FN adsorbed onto surfaces from solutions with concentrations ranging from 1 to 500 µg ml^−1^ in DI water, following a single wash. (D) line plot showing fluorescence intensity of adsorbed BODIPY-FN at various concentrations after washing; pink dots represent three technical replicates, blue dots indicate average values, and the red dotted line marks the di water control.

Fibronectin was first concentrated, and tris amine was removed from the TBS buffer prior to conjugation to minimize nonspecific labeling. Following conjugation with BODIPY, excess unbound dye and buffer salts were removed through extensive washing (Fig. 2A). BODIPY-FN was then lyophilized to determine its dry mass, enabling accurate concentration calculation (Fig. 2B). BODIPY-FN was serially diluted across a concentration range of 1 to 500 μg mL^−1^, incubated in a 96-well plate for 3 hours, washed with 1× PBS, and imaged using fluorescence microscopy (Fig. 2C). Fluorescence intensity was subsequently quantified using a plate reader (Fig. 2D and Fig. S3D). The results showed a concentration-dependent increase in adsorbed BODIPY-FN, with a detectable signal observed at concentrations of 10 μg mL^−1^ or higher.

**Fig. 3:**
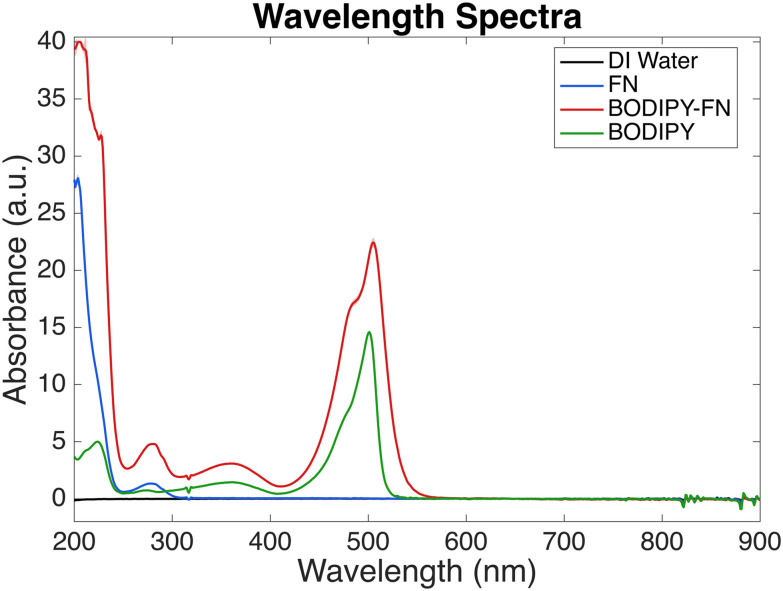
UV–Vis spectra of DI water, fibronectin (FN), BODIPY, and the BODIPY-fibronectin (BODIPY-FN) conjugate, used to determine the degree of BODIPY labeling on FN. Each condition was measured in triplicate, and the averaged spectra are shown as colored lines with standard deviations indicated by the corresponding shaded regions.

The degree of labeling (DOL) for BODIPY-FN was determined by UV–Vis spectroscopy using eqn (1), which incorporates absorbance at 280 nm and the fluorophore maximum at 504 nm (Fig. 3). Triplicate measurements yielded a DOL of 27.35% ± 0.07, indicating consistent and efficient incorporation of BODIPY into fibronectin. This labeling level provided sufficient signal for both fluorescence-based characterization and subsequent fluorine-based surrogate labeling analysis, while remaining low enough to minimize potential effects on fibronectin adsorption or structural integrity.

**Fig. 4:**
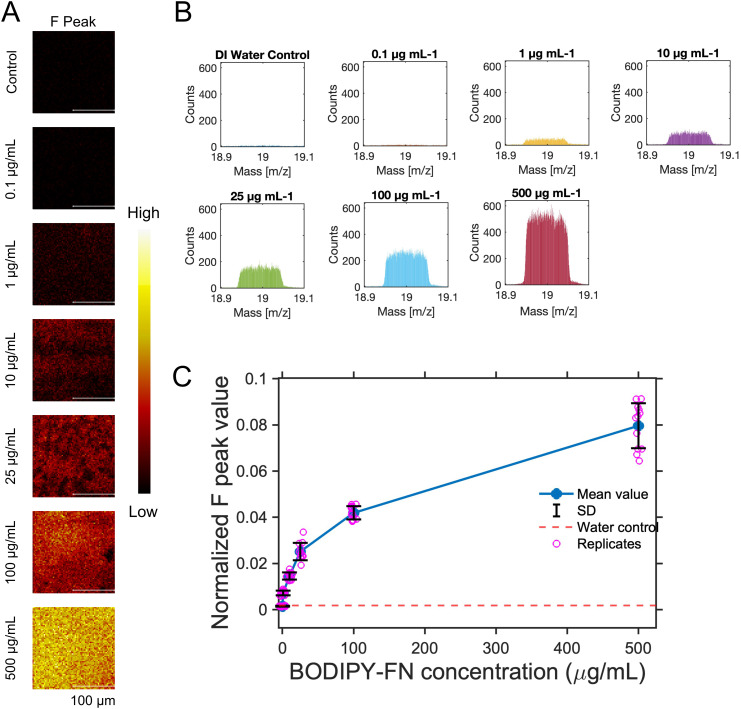
ToF-SIMS analysis of varying concentrations of BODIPY-fibronectin (BODIPY-FN) coated on plastic slide surfaces. (A) Representative ToF-SIMS images showing fluorine (F) ion signals (*m*/*z* 18.898–19.098) for BODIPY-FN coatings ranging from 0.1 to 500 μg ml^−1^, with water as a control. (B) F ion peak at *m*/*z* 18.998 across different BODIPY-FN concentrations, displayed using a uniform *y*-axis scale. (C) Normalized f ion counts, calculated by dividing F ion counts by total ion counts for each ROI, presented as scatter points in a line plot. The water control is indicated by a red dotted line, mean values are shown as blue dots, standard deviations are shown in black, and pink dots represent 12 ROIs measured from three distinct locations. All measurements were acquired in unbunched mode.

For the BODIPY-FN measurements using ToF-SIMS, BODIPY-FN was prepared at concentrations ranging from 0.1 to 500 μg mL^−1^ in DI water and applied to plastic slides to generate a standard curve. DI water was used instead of 1× PBS to minimize background interference from sodium and potassium salts, which could obscure the fluorine signal in mass spectrometry analysis. Following overnight incubation and rinsing with DI water, the samples were analyzed by ToF-SIMS in negative ion mode. Representative fluorine [F]^−^ ion maps are shown in Fig. 4A, along with corresponding F ion spectra across the concentration range (Fig. 4B). Normalized F ion counts were calculated (Table S3) and plotted against BODIPY-FN concentration, revealing a logarithmic increase in signal intensity with increasing concentration (Fig. 4C and Fig. S4A). The data points across both the full concentration range (0.1–500 µg mL^−1^) and the lower concentration range (0.1–25 µg mL^−1^) fit well to a power law, exhibiting high *R*-squared values close to 1 (Fig. S4). Concentrations of 1 µg mL^−1^ or higher were clearly distinguishable from the DI water control (Fig. S3A). Based on its high sensitivity and low background signal in ToF-SIMS measurement, BODIPY-FN was selected for subsequent ligand gradient experiments.

#### 3.1.4. BODIPY-FN gradient generation in microfluidic chips and ToF-SIMS characterization

Microfluidic chips have been widely used to generate ligand gradients for studying cellular responses to both soluble (chemotaxis) and insoluble (haptotaxis) cues.^6–8^ Our group previously demonstrated the use of a multiplex microfluidic platform to create diffusion-based concentration gradients for haptotaxis studies.^4^ In this study, the same microfluidic system was used to generate a diffusion-based BODIPY-FN gradient using source chamber concentrations of 100 and 500 µg mL^−1^, along with a water control (Fig. S5A). The gradient was formed over 24 hours of diffusion and confirmed by averaging line profiles across multiple chambers (Fig. S5B). After the gradient was established, the device was washed with DI water to remove any excess reagent. The flexible plastic slide was then detached from the PDMS housing, revealing the surface-adsorbed BODIPY-FN (Fig. S6A). The slide was subsequently sectioned into smaller pieces and mounted for ToF-SIMS analysis to measure the surface F distribution along the direction of BODIPY-FN diffusion (Fig. S6B and S6C).

**Fig. 5:**
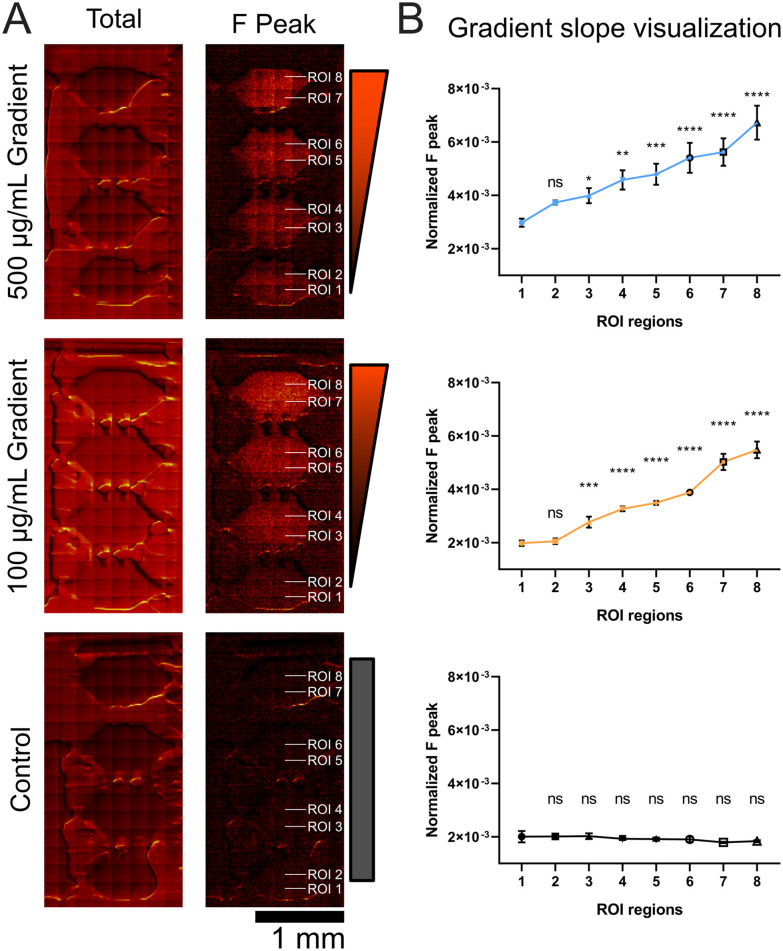
BODIPY-FN gradient formation within microfluidic chambers as measured by ToF-SIMS imaging. (A) ToF-SIMS mosaic images (composed of 200 µm by 200 µm images) of four-chamber units showing BODIPY-FN diffusion from the top to bottom chambers, resulting in gradient formation using 500 µg ml^−1^ BODIPY-FN (top panel), 100 µg ml^−1^ BODIPY-FN (middle panel), or water control (bottom panel). Both total ion and fluorine (F) ion ToF-SIMS images are displayed side by side. Rectangular ROIs, with their relative locations indicated by white text on the images, were drawn for analysis of normalized f peak counts. (B) Line plot of normalized F peak counts for different ROI regions shows the BODIPY-FN gradient. Each region was sampled with three ROIs, and the mean and standard deviation were calculated. Statistical comparisons were made against the ROI 1 region using one-way ANOVA with a *post hoc* Dunnett test. ns: not significant; *p < 0.05; **p < 0.01; ***p < 0.001; ****p < 0.0001.

BODIPY-FN gradients generated from source chamber concentrations of 500 or 100 µg mL^−1^ were mapped using ToF-SIMS and compared to a water-only control (Fig. 5A). Both total ion and F ion images were acquired, with the F^−^ distribution visualized as a heat map showing a decreasing gradient along the *y*-axis, corresponding to the direction of diffusion. Within each microfluidic chamber, multiple rectangular regions of interest (ROIs) were defined along the diffusion axis and averaged across the *x*-axis to quantify F signal intensity (Fig. 5B). Both 100 µg mL^−1^ and 500 µg mL^−1^ conditions produced detectable gradients with distinct slopes and corresponding concentration profiles, while the water control showed uniformly low F signal throughout the chamber.

**Fig. 6:**
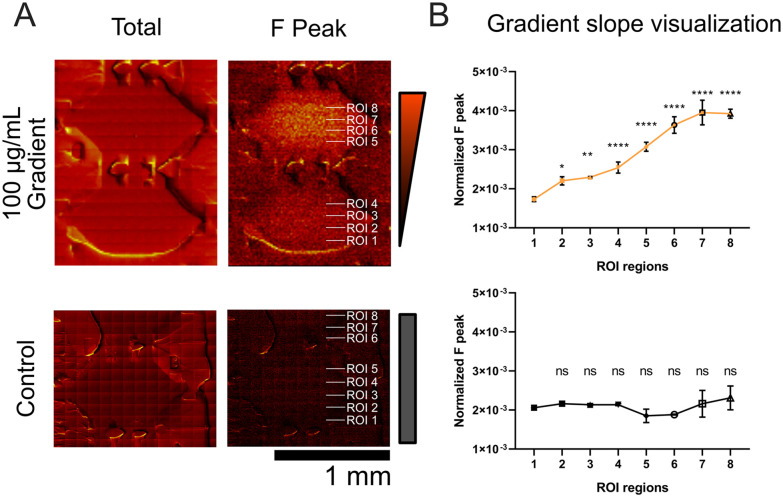
BODIPY-FN gradient formation (100 µg ml^−1^) within lower microfluidic chambers measured by ToF-SIMS imaging. (A) ToF-SIMS mosaic images (100 µm by 100 µm) of two-chamber units showing gradients from BODIPY-FN (top panel) or water control (bottom panel), with total ion and fluorine (F) ion images side by side. Rectangular ROIs, with their relative locations indicated by white text on the images, were drawn for analysis of normalized f peak counts. (B) Line plot of normalized F peak counts for different ROI regions shows the BODIPY-FN gradient. Each region was sampled with three ROIs, and the mean and standard deviation were calculated. Statistical comparisons were made against the ROI 1 region using one-way ANOVA with a *post hoc* dunnett test. ns: not significant; *p < 0.05; **p < 0.01; ***p < 0.001; ****p < 0.0001.

The BODIPY-FN gradient was clearly detectable within individual chambers. We further examined the lower chambers (chambers A and B) of the 100 µg mL^−1^ condition to determine whether higher spatial resolution and longer acquisition time would improve gradient visualization. Chambers A and B were imaged using mosaic mode with a finer step size of 100 µm × 100 µm, capturing both total ion and F ion distributions (Fig. 6A). Four rectangular ROI regions were defined within each chamber to analyze the F gradient at a finer scale. Line plots of the F ion signal revealed a clear gradient within individual chambers, in contrast to the water control, which showed uniformly low F levels (Fig. 6B). Quantification in the top chamber (chamber B) demonstrates the spatial gradient distribution corresponding to the haptotaxis range previously reported in our earlier study, which was challenging to capture using fluorescence-based methods.^4^ Overall, BODIPY-FN exhibited the lowest background signal, enabling fluorescence-based detection of fibronectin above 10 µg mL^−1^ and achieving even greater sensitivity with ToF-SIMS down to 1 µg mL^−1^. These results, along with the demonstration of gradient measurement in ToF-SIMS, support the use of BODIPY-FN as a surrogate marker, providing high spatial resolution and sensitivity for mapping surface-adsorbed protein distributions.

### 3.2. Discussion

Haptotactic guidance by extracellular matrix (ECM) proteins such as fibronectin (FN) is essential for various biological processes, including embryogenesis, wound healing, and immune responses.^1,2,24^ Although plasma fibronectin concentration in human serum has been reported at approximately 300 μg mL^−1^, accurately measuring cellular fibronectin concentration *in vivo* remains challenging.^25^ Feng *et al.* demonstrated a spatial gradient of fibronectin in the developing mouse skull using immunostaining, and provided evidence that this distribution guides calvarial osteoblast progenitor cells toward the skull roof.^24^ However, quantifying fibronectin concentration based on fluorescence intensity is limited by technical constraints. *In vitro*, microfluidic platforms are widely used to study haptotaxis, with gradient visualization typically relying on high concentrations of fluorescently labeled proteins or surrogates such as dextran.^4,6,7^ These methods often lack the sensitivity to detect the low, physiologically relevant ligand densities where cells respond, making it difficult to accurately define the microenvironment. Improved sensitivity is essential for capturing these subtle gradients and cell–matrix interactions to better inform biomaterial design. As expected with its expansive application, fibronectin concentrations used in *in vitro* studies vary broadly, ranging from 2 μg mL^−1^ to 500 μg mL^−1^ depending on the experimental objectives, cell types, and surface coating conditions.^4,6,26,27^ This wide range of experimental protein gradients highlights the need for alternative methods beyond fluorescence-based detection, which is only sensitive enough to capture the higher concentration range (*e.g.*, FITC-dextran at 100 µg mL^−1^). Such an ideal method would have the ability to accurately measure and visualize fibronectin at the lower concentrations approaching the limits of traditional fluorescent microscopy where migration physiologically occurs.

In this study, we initially determined the limit of detection for FITC-dextran within a microfluidic chip and then evaluated the feasibility of using ToF-SIMS for gradient detection. To explore suitable surrogates for mapping fibronectin gradients using ToF-SIMS, we developed and evaluated several standard approaches, including BCA assays, Eosin staining, and a novel BODIPY-conjugated fibronectin. These standards were tested across varying fibronectin concentrations ranging from 1 to 500 µg mL^−1^. The BODIPY-conjugated fibronectin provided dual functionality, enabling both fluorescent detection of fibronectin and elemental analysis *via* ToF-SIMS due to its fluorine tag. In comparison, elemental signals from copper (Cu) in BCA and bromine (Br) in Eosin exhibited varying background levels, allowing reliable detection of fibronectin at concentrations ≥100 µg mL^−1^ and ≥10 µg mL^−1^, respectively. In contrast, BODIPY-FN demonstrated the lowest background signal, permitting fluorescence-based detection of fibronectin at 10 µg mL^−1^ or higher and enabling even greater sensitivity with ToF-SIMS detection down to 1 µg mL^−1^. Furthermore, we successfully demonstrated the generation and visualization of diffusion-based gradients within a two-layer microfluidic chip. Following removal of the PDMS layer, fluorine elemental analysis by ToF-SIMS directly revealed the spatial distribution of the fibronectin ligand gradient. These results highlight the potential of ToF-SIMS as a highly sensitive, semi-quantitative technique for characterizing fibronectin gradients, and suggest that, with proper standardization, accurate quantification of a wide range of fibronectin concentrations can be achieved.

BCA assay and Eosin staining are widely used to detect total protein content and can be applied to assess the presence of all proteins on a surface. In our study, we demonstrated that these non-specific detection methods can serve as effective surrogates for ToF-SIMS analysis by identifying surface-adsorbed fibronectin (FN) across a range of known concentrations. While suitable for characterizing single-ligand systems, this approach lacks the specificity required to distinguish between multiple extracellular matrix (ECM) ligands. To overcome this limitation, we used BODIPY-FN, which enabled ligand-specific detection compatible with ToF-SIMS. In more complex systems containing multiple ligands such as collagen, vitronectin, and laminin, additional tagging strategies are necessary to differentiate each component. Surrogate labeling has been shown in previous studies to be an effective solution. For example, 15N-labeling produces distinct C15N fragment ions, allowing precise ToF-SIMS mapping of 15N-labeled proteins on micropatterned surfaces.^28^ Another study demonstrated the use of an iodine-containing probe that incorporates both a fluorophore for fluorescence detection and a unique 127I signal detectable by ToF-SIMS, enabling dual-mode imaging of target proteins.^29^ By applying such isotope or surrogate labeling strategies, it becomes possible to detect and quantify multiple ligand concentration gradients on the same surface with high spatial resolution.

During ToF-SIMS analysis, potential sources of contamination must be carefully considered to ensure accurate surface characterization. One factor that requires attention is the PDMS material used in the fabrication of the microfluidic chip. To expose the plastic slide containing the ligand gradient, the PDMS chamber and roofing were removed either by peeling or by physically cutting with a razor blade. However, residual PDMS may remain on the surface and produce characteristic fragment peaks in the mass spectrum. These fragments can interfere with elemental analysis, particularly in positive ion mode, where peaks may appear at *m*/*z* 28, 43, 73, 133, 147, 207, 221, and 281,^30^ potentially masking nearby analyte signals. Buffer salts, such as those from phosphate-buffered saline (PBS) or Tris-buffered saline (TBS), also need to be considered. Upon drying, these salts may crystallize on the surface and complicate spectrum interpretation. In our preliminary data, fibronectin was diluted in 1× PBS, which resulted in relatively strong sodium (*m*/*z* 23) and potassium (*m*/*z* 39) signals. These peaks can contribute to background noise and interfere with the detection of nearby target signals. Therefore, DI water was used in place of 1× PBS to minimize salt-derived background and prevent masking of relevant peaks. In addition, TBS, the original buffer for the fibronectin stock, can interfere with BODIPY conjugation chemistry. To address this issue, an additional desalting step using a column was performed to remove Tris and prevent unwanted interactions during the labeling process. These considerations emphasize the importance of careful buffer selection and surface preparation to reduce background signals and improve the reliability of ToF-SIMS analysis.

Although ToF-SIMS offers numerous advantages for measuring ligand gradients using surrogate tagging, such as high surface sensitivity, excellent spatial resolution down to 100 nanometers, strong chemical specificity, and the ability to perform both two-dimensional and three-dimensional mapping through depth profiling, several technical challenges must still be considered. In this study, the issue of extracellular matrix protein fragmentation was addressed by conjugating ligands with a BODIPY-based surrogate and using fluorine as the detectable element. The unique fluorine peak remained clearly detectable with minimal background noise, even when using slightly lower mass resolution in spatial imaging mode. One of the primary limitations of ToF-SIMS is its semi-quantitative nature, as signal intensities represent relative rather than absolute concentrations. As a future direction, incorporating internal standards within the chip design may enable more accurate quantification of ligand gradients across the surface. Additionally, potential surface contamination remains a source of variability that must be managed carefully. The extreme surface sensitivity of ToF-SIMS, which generally probes the top 2–3 nm, can make it difficult to detect differences in ligand concentration when the surface is saturated. However, depth profiling through sputtering offers a way to evaluate ligand distribution at higher concentrations. Prolonged measurement over gradient regions can also result in sample degradation due to continuous ion bombardment, which reduces signal stability and limits the feasibility of repeated scans. Using ToF-SIMS, we observe a surrogate element gradient across the chip down to the lowest concentration range below limits of detection achieved with fluorescence for all groups (100, 500 µg ml^−1^). The observation that different concentrations achieved similar values (with a discernible shift down from 500 to 100) was intriguing. We anticipate the difference results from a few factors. The ToF-SIMS analysis is a surface analysis and for the source chamber of the chip, a saturated plane of fibronectin (as observed by fluorescence) will quantitatively appear the same. We have observed that increasing the scan time to allow for etching can demonstrate volumetric differences in surrogate element when detecting multiple layers of fibronectin. At the lower concentrations of fibronectin along the gradient, the probability of secondary ions reaching the detector decreases with fewer fluorines available due to fewer fibronectin proteins, random orientation of the proteins, and increasing contribution of the plastic substrate on ionization potential with lower density of proteins.

In addition to ToF-SIMS, several other analytical techniques may be considered for future studies to provide complementary insights into ligand gradient characterization. X-ray Photoelectron Spectroscopy (XPS) has been used to analyze laminin-derived peptide gradients designed to guide Schwann cell migration,^31^ as well as fibronectin and VEGF gradients for directing endothelial cell migration.^32^ While a comparative study has demonstrated that ToF-SIMS offers superior detection limits and spatial resolution for detecting low amounts of adsorbed proteins,^15^ XPS provides more reliable quantitative information, making it advantageous for determining absolute surface coverage.^33^ Surface plasmon resonance (SPR) is another valuable technique that has been applied to quantify adsorption kinetics and ligand gradients in combination with other surface characterization methods. For example, fibronectin gradients immobilized on gold were validated using both SPR and XPS,^34^ and SPR has also been used to measure the surface-bound mass of proteins such as fibronectin and VEGF.^32^ Furthermore, SPR imaging has been used to monitor dynamic interactions between live cells and extracellular matrix proteins, as demonstrated in a study observing real-time fibronectin deposition by vascular smooth muscle cells.^35^ Together, ToF-SIMS and SPR offer complementary strengths, with ToF-SIMS excelling in high-resolution static mapping of ligand distribution and SPR providing dynamic and functional measurements of binding events. Another mass spectrometry-based technique, matrix-assisted laser desorption/ionization mass spectrometry (MALDI-MS), has been applied to confirm gradient formation in photoresponsive amyloid-like RGD epitope fibril surfaces.^36^ While ToF-SIMS offers higher spatial resolution for small molecules and tagged ligands, MALDI-MS is better suited for analyzing the distribution of intact large proteins and peptides. Finally, atomic force microscopy (AFM) provides nanoscale spatial resolution and the capability to probe surface topography and mechanical properties, making it especially useful for assessing local variations in ligand presentation. Although AFM has not yet been applied to directly map fibronectin concentration gradients, it has been successfully used to quantify fibronectin-integrin interactions,^37^ and visualize early stages of fibronectin fibrillogenesis in living cells.^38^ Incorporating these techniques in future work may significantly enhance the understanding of how spatial variations in ligand density influence molecular interactions, signaling, and cell behavior.

## 4. Conclusions

In conclusion, this study establishes ToF-SIMS as a sensitive and high-resolution method for characterizing fibronectin gradients on synthetic surfaces commonly used in *in vitro* cell migration assays. By labeling fibronectin with distinct surrogate elements such as copper, bromine, or fluorine, we achieved semi-quantitative measurements of fibronectin concentration. By conjugating fibronectin with BODIPY, we created a dual-functional ligand that enabled fluorescence-based detection and elemental analysis *via* ToF-SIMS through its fluorine tag. Compared to copper in BCA and bromine in Eosin, which showed higher background and required concentrations ≥100 µg mL^−1^ or ≥10 µg mL^−1^ for reliable detection, respectively, BODIPY-fibronectin offered the lowest background, allowing fluorescence detection at ≥10 µg mL^−1^ and extending ToF-SIMS sensitivity down to 1 µg mL^−1^. Using a microfluidic chip, we successfully created a BODIPY-fibronectin gradient and confirmed the applicability of ToF-SIMS for detecting ligand gradients labeled with fluorine surrogate. This strategy can be extended to multiple ligands, providing a reliable and versatile approach for future quantitative measurements of ligand distributions in studies of cell migration and related processes.

## Author contributions

C. L.: conceptualization, data curation, investigation, methodology, validations, formal analysis, visualization, software, writing – original draft; T. K.: investigation, data curation, methodology, validations, formal analysis, writing – review & editing; D. W.: investigation, data curation, methodology, validations, writing – review & editing; R. A.: funding acquisition, resources, writing – review & editing; R. S.: funding acquisition, resources, writing – review & editing; S. S.: conceptualization, data curation, project administration, funding acquisition, resources, supervision, writing – review & editing.

## Conflicts of interest

There are no conflicts to declare.

## Supplementary Material

AN-151-D5AN00962F-s001

AN-151-D5AN00962F-s002

AN-151-D5AN00962F-s003

AN-151-D5AN00962F-s004

AN-151-D5AN00962F-s005

AN-151-D5AN00962F-s006

AN-151-D5AN00962F-s007

AN-151-D5AN00962F-s008

## Data Availability

The data supporting this article have been included as part of the supplementary information (SI). Supplementary information: ToF-SIMS measurements of Cu, Br, and F surrogates for fibronectin standards (Tables S1–S3), ToF-SIMS analysis of varying concentration of fibronectin using BCA (Fig. S1) or Eosin (Fig. S2) as surrogate marker, statistical analysis (Fig. S3), line fitting for BODIPY-FN standards (Fig. S4), visualization of the BODIPY-FN gradient by fluorescence (Fig. S5), and the setup of the microfluidic chip with ToF-SIMS measurement (Fig. S6). See DOI: https://doi.org/10.1039/d5an00962f.
