# Supplementary material for: Evaluation of ToF-SIMS imaging for semi-quantitative mapping of BODIPY-labeled fibronectin surface gradients
Source: Analyst. 2026 Jan 19;151(4):1118–29. doi: 10.1039/d5an00962f (PMC12814933; doi:10.1039/d5an00962f)
Supplement: AN-151-D5AN00962F-s001 [file AN-151-D5AN00962F-s001.pdf]

Supplementary Information (SI) for Analyst.
This journal is © The Royal Society of Chemistry 2026

Supplementary information for:

Evaluation of ToF-SIMS imaging for semi-quantitative mapping of BODIPY-labeled fibronectin surface gradients

Chao Liu,[a] Tae Kyong John Kim, [b] Douglas H. Wu,[a,c] Radhika Atit,[d] Rodrigo A. Somoza,[e,f] and Samuel E. Senyo[a]*

a. Department of Biomedical Engineering, Case Western Reserve University, Cleveland, OH, USA.

b. Swagelok Center for Surface Analysis of Materials, Case Western Reserve University, Cleveland, OH, USA.

c. Medical Scientist Training Program, Case Western Reserve University, Cleveland, OH, USA.

d. Department of Biology, Case Western Reserve University, Cleveland, OH, USA.

e. Department of Biology, Skeletal Research Center, Case Western Reserve University, Cleveland, OH, USA.

f. Center for Modular Manufacturing of Structural Tissues, Case Western Reserve University, Cleveland, OH, USA

Table S 1. Summary of total counts, Cu counts, and normalized values for each condition in BCA-stained fibronectin standards. Data are presented as mean ± standard deviation from four regions of interest (ROIs), each with an approximate scanning size of 100 µm × 100 µm.

Condition	Total Count (mean ± SD)	Cu Count (mean ± SD)	Normalized Value (mean ± SD)
Control	174,655 ± 63,720	2,141 ± 489	1.28×10^{-2} ± 1.98×10^{-3}
1 µg/mL FN	171,636 ± 14,044	1,854 ± 154	1.08×10^{-2} ± 1.71×10^{-4}
10 µg/mL FN	168,117 ± 20,139	2,122 ± 176	1.27×10^{-2} ± 5.97×10^{-4}
100 µg/mL FN	163,730 ± 12,637	3,047 ± 181	1.86×10^{-2} ± 8.77×10^{-4}

Table S 2. Summary of total counts, Br counts, and normalized values for each condition in Eosin-labeled fibronectin standards. Data are presented as mean ± standard deviation from four regions of interest (ROIs), each with an approximate scanning size of 100 µm × 100 µm.

Condition	Total Count (mean ± SD)	Br Count (mean ± SD)	Normalized Value (mean ± SD)
Control	362,472 ± 44,971	205 ± 137	5.43×10^{-4} ± 3.11×10^{-4}
1 µg/mL FN	356,600 ± 54,962	287 ± 82	8.03×10^{-4} ± 1.77×10^{-4}
10 µg/mL FN	317,606 ± 46,686	491 ± 77	1.54×10^{-3} ± 2.85×10^{-5}
100 µg/mL FN	293,649 ± 46,133	2,272 ± 448	7.70×10^{-3} ± 4.48×10^{-4}

Table S 3. Summary of total counts, F counts, and normalized values for each condition in BODIPY conjugated fibronectin standards. Data are presented as mean ± standard deviation from four regions of interest (ROIs), each with an approximate scanning size of 100 µm × 100 µm.

Condition	Total Count (mean ± SD)	F Count (mean ± SD)	Normalized Value (mean ± SD)
Control	360,695 ± 39,051	654 ± 74	1.83×10^{-3} ± 2.94×10^{-4}
0.1 µg/mL FN	373,663 ± 80,307	564 ± 172	1.49×10^{-3} ± 1.62×10^{-4}
1 µg/mL FN	371,459 ± 23,260	2,270 ± 467	7.30×10^{-3} ± 1.02×10^{-3}
10 µg/mL FN	359,560 ± 49,511	5,297 ± 1,112	1.46×10^{-2} ± 1.57×10^{-3}
25 µg/mL FN	364,388 ± 37,795	9,156 ± 1,646	2.51×10^{-2} ± 3.77×10^{-3}
100 µg/mL FN	363,236 ± 57,319	15,291 ± 3,040	4.19×10^{-2} ± 2.87×10^{-3}
500 µg/mL FN	485,247 ± 137,104	38,067 ± 10,209	7.96×10^{-2} ± 9.74×10^{-3}

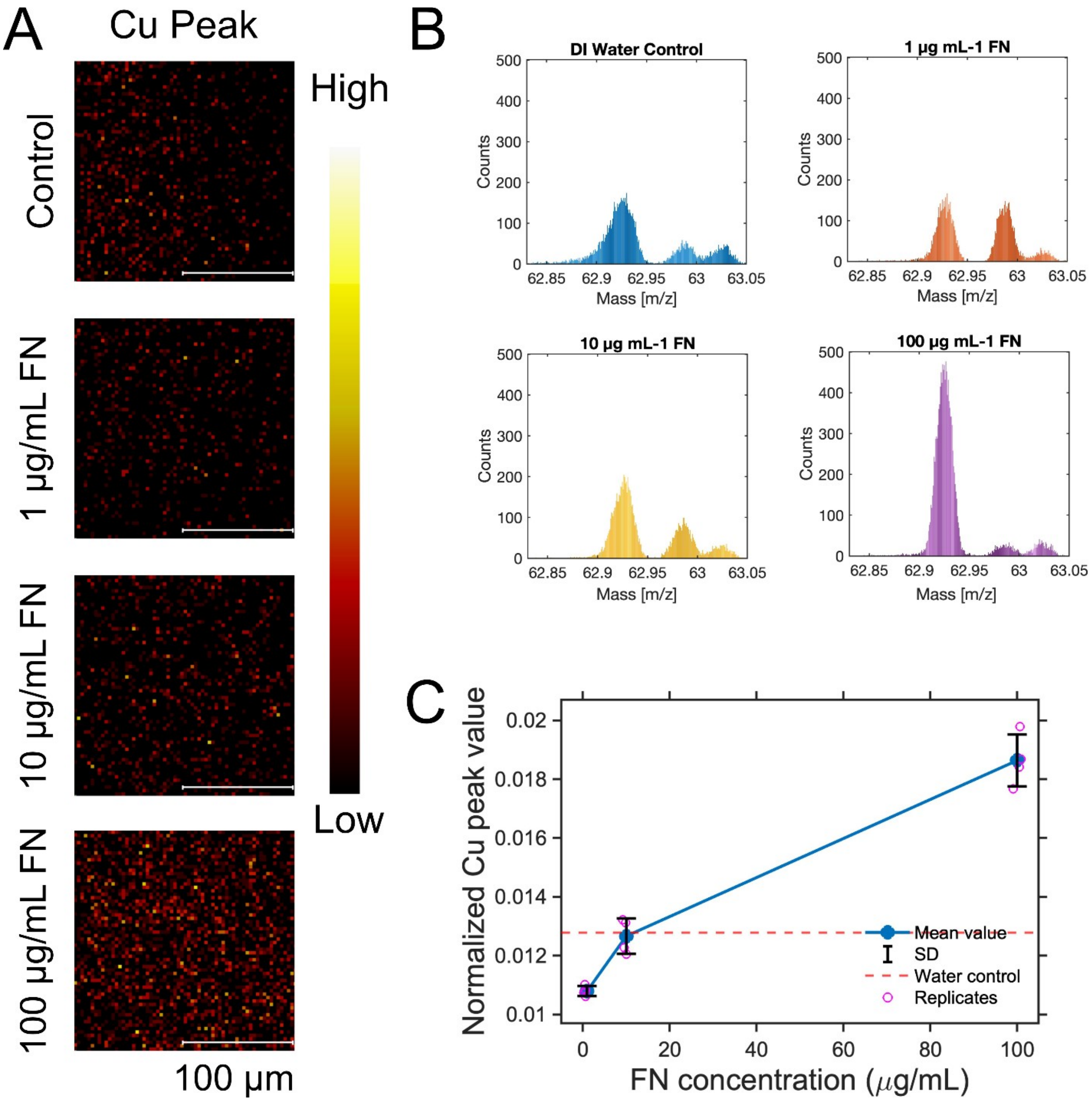

Supplementary Figure 1. ToF-SIMS analysis of varying concentrations of fibronectin (FN) coated onto plastic slide surfaces, using BCA staining as a surrogate marker. **(A)** Representative ToF-SIMS images illustrating copper (Cu) ion signals (m/z 62.83–63.03) across fibronectin (FN) coatings ranging from 1 to 100 µg mL-1, with water as a control. **(B)** Cu ion peak intensities at m/z 62.93 for different FN concentrations, displayed using a uniform y-axis scale for comparison. **(C)** Normalized Cu ion counts, calculated by dividing Cu ion counts by total ion counts for each ROI, presented as pink scatter points in a line plot. The water control is indicated by a red dotted line, mean values are shown as blue dots, standard deviations are shown in black, and pink dots represent 4 ROIs. Measurements were acquired in bunched mode.

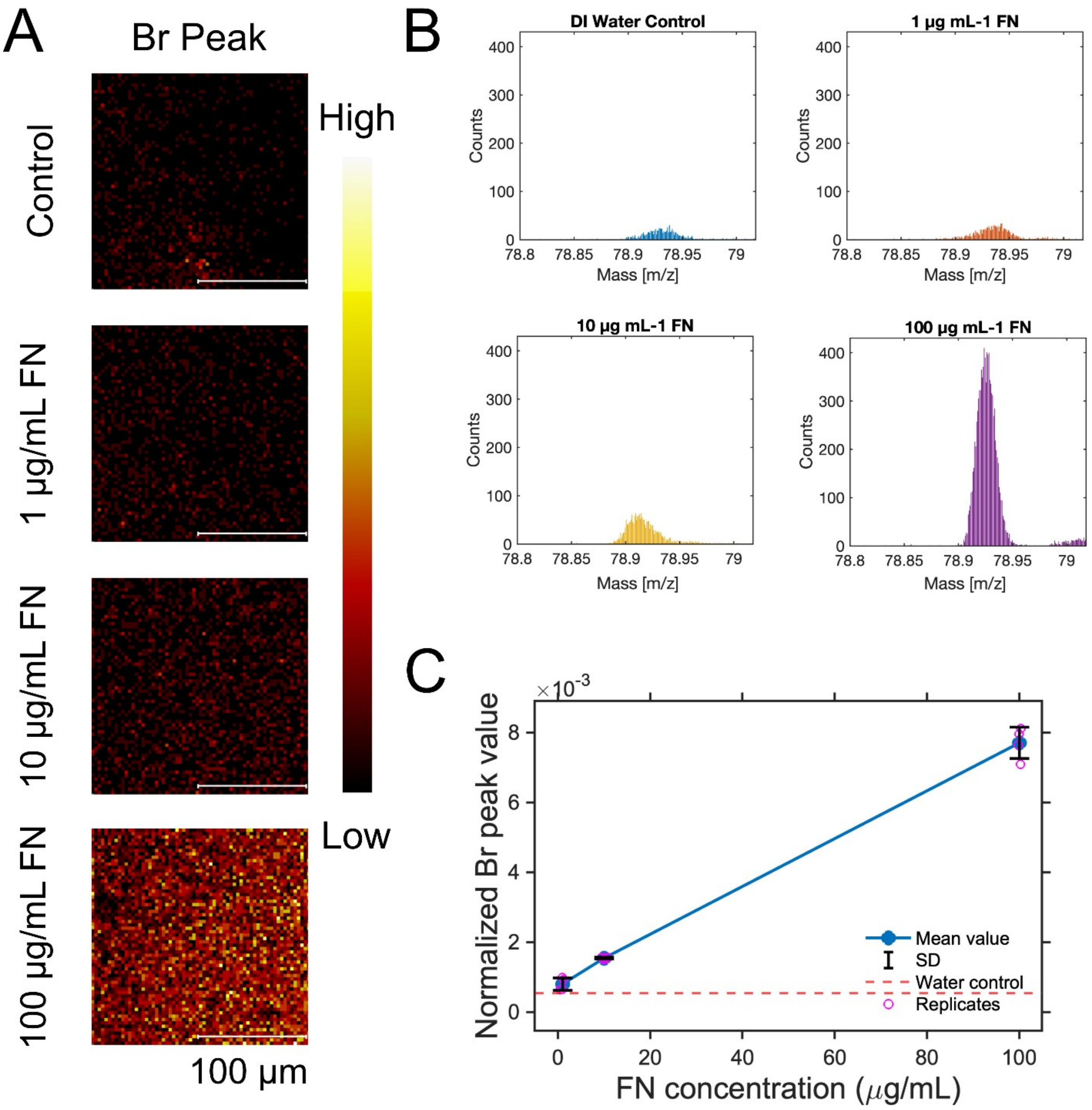

Supplementary Figure 2. ToF-SIMS analysis of varying concentrations of fibronectin (FN) coated onto plastic slide surfaces, using Eosin staining as a surrogate marker. **(A)** Representative ToF-SIMS images illustrating bromine (Br) ion signals (m/z:78.818-79.018) across FN coatings ranging from 1 to 100 μg mL-1, with water as a control. **(B)** Br ion peak at m/z 78.92 for different FN concentrations displayed using a uniform y-axis scale. **(C)** Normalized Br ion counts, calculated by dividing Br ion counts by total ion counts for each ROI, presented as scatter points in a line plot. The water control is indicated by a red dotted line, mean values are shown as blue dots, standard deviations are shown in black, and pink dots represent 4 ROIs. Measurements were acquired in bunched mode.

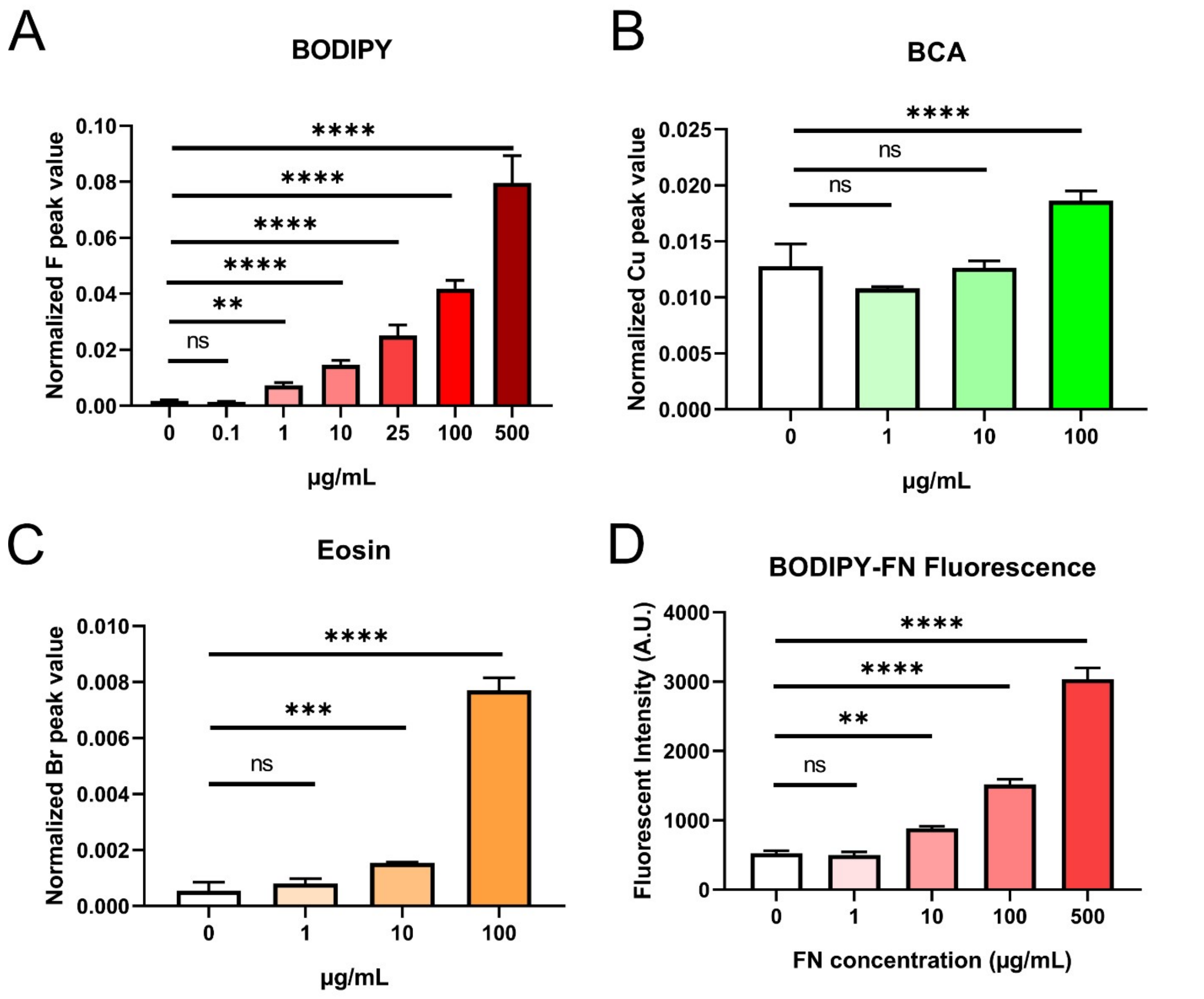

Supplementary Figure 3. ToF-SIMS analysis of varying concentrations of BODIPY–FN **(A)**, fibronectin labeled with surrogate BCA **(B)**, fibronectin labeled with Eosin **(C)**, and fluorescence measurements of BODIPY–FN at corresponding concentrations **(D)**. Statistical comparisons were made against the water control (0 μg mL^{-1}) using one-way ANOVA with a post hoc Dunnett test. ns: not significant; *$p < 0.05$; **$p < 0.01$; ***$p < 0.001$; ****$p < 0.0001$.

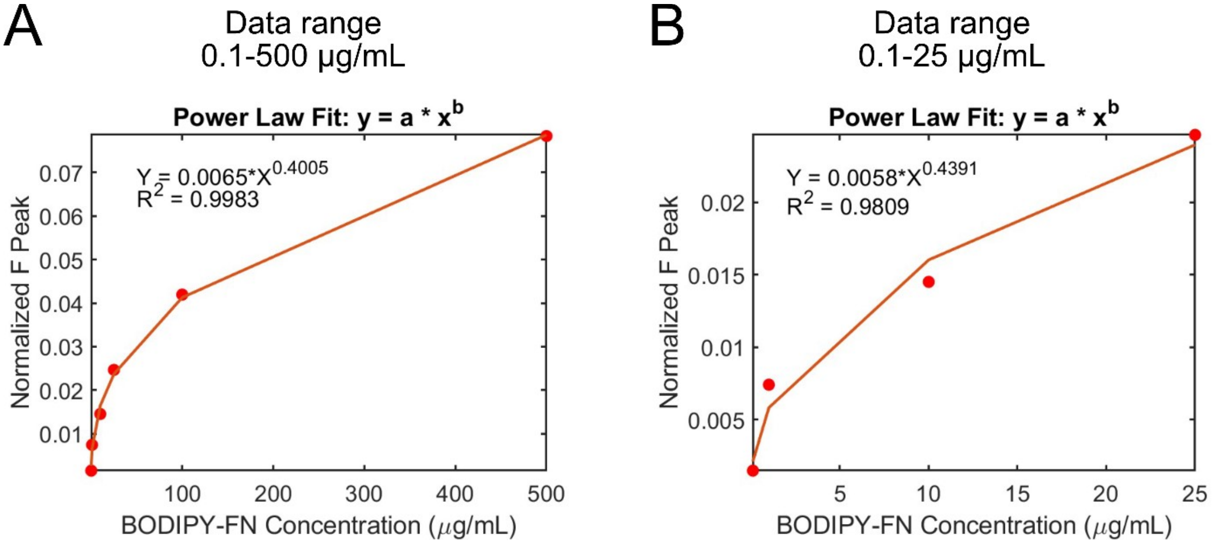

Supplementary Figure 4. Power law fits were applied to the BODIPY-FN standard concentrations for **(A)** data in the range of 0.1–500 µg mL^{-1} and **(B)** data in the range of 0.1–25 µg mL^{-1}. The fitting equations and R-squared values are provided on the graphs.

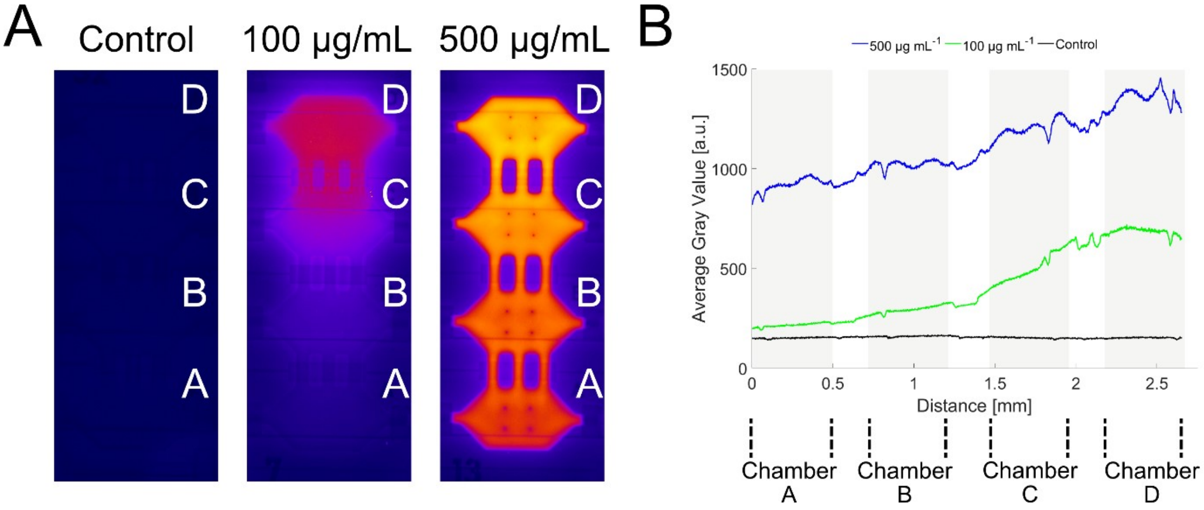

Supplementary Figure 5. BODIPY-FN gradient formation in the microfluidic chip visualized by fluorescence imaging. (A) Fluorescence images of BODIPY-FN gradients formed by 24-hour diffusion from source chambers containing 100 µg mL^{-1} or 500 µg mL^{-1} BODIPY-FN, compared to the water control. (B) Averaged line profiles of fluorescence intensity across chambers A–D, demonstrating gradient formation.

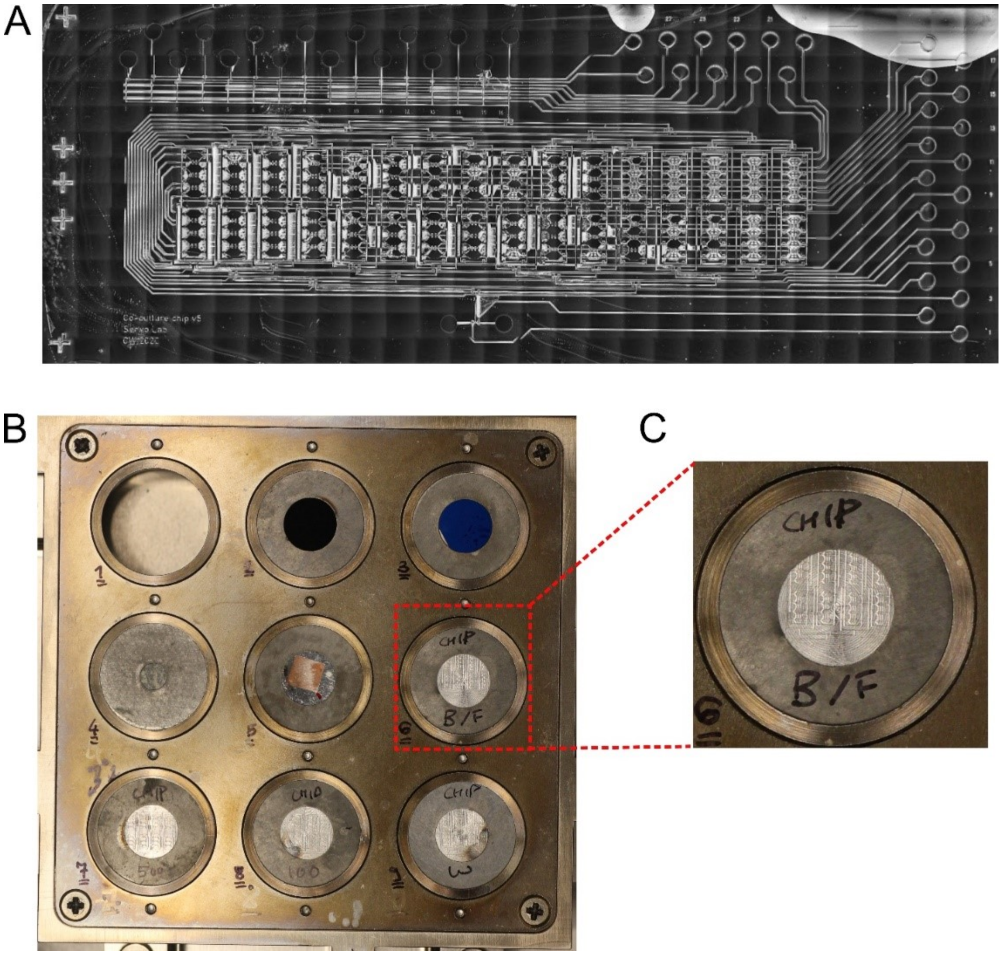

Supplementary Figure 6. Images of the microfluidic chip used to generate BODIPY-FN gradients and prepare samples for ToF-SIMS analysis. **(A)** Overview of the microfluidic chip after removal of the PDMS chamber, revealing the underlying plastic surface. **(B)** Sample plate showing the microfluidic slide cut into smaller pieces for ToF-SIMS measurement. **(C)** Close-up image of the microfluidic chambered slide used for ToF-SIMS analysis.
