# Supplementary figures and images for: Evaluation of ToF-SIMS imaging for semi-quantitative mapping of BODIPY-labeled fibronectin surface gradients
Source: Analyst. 2026 Jan 19;151(4):1118–29. doi: 10.1039/d5an00962f (PMC12814933; doi:10.1039/d5an00962f)

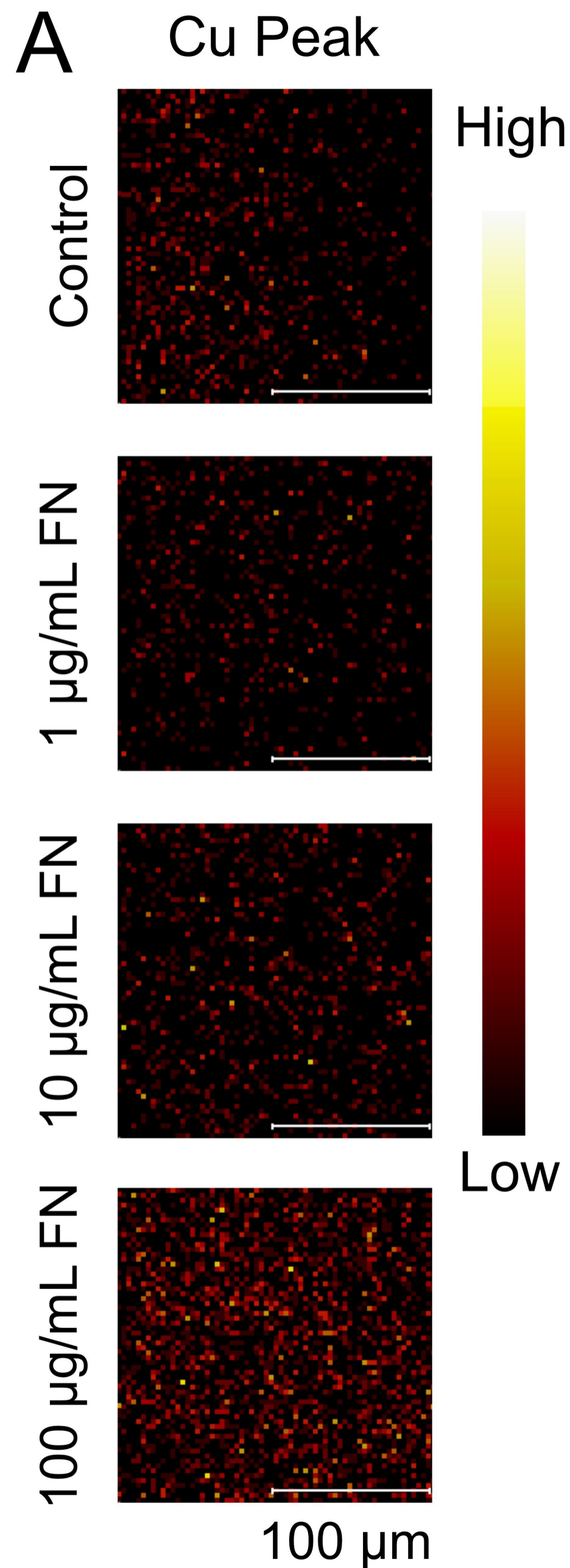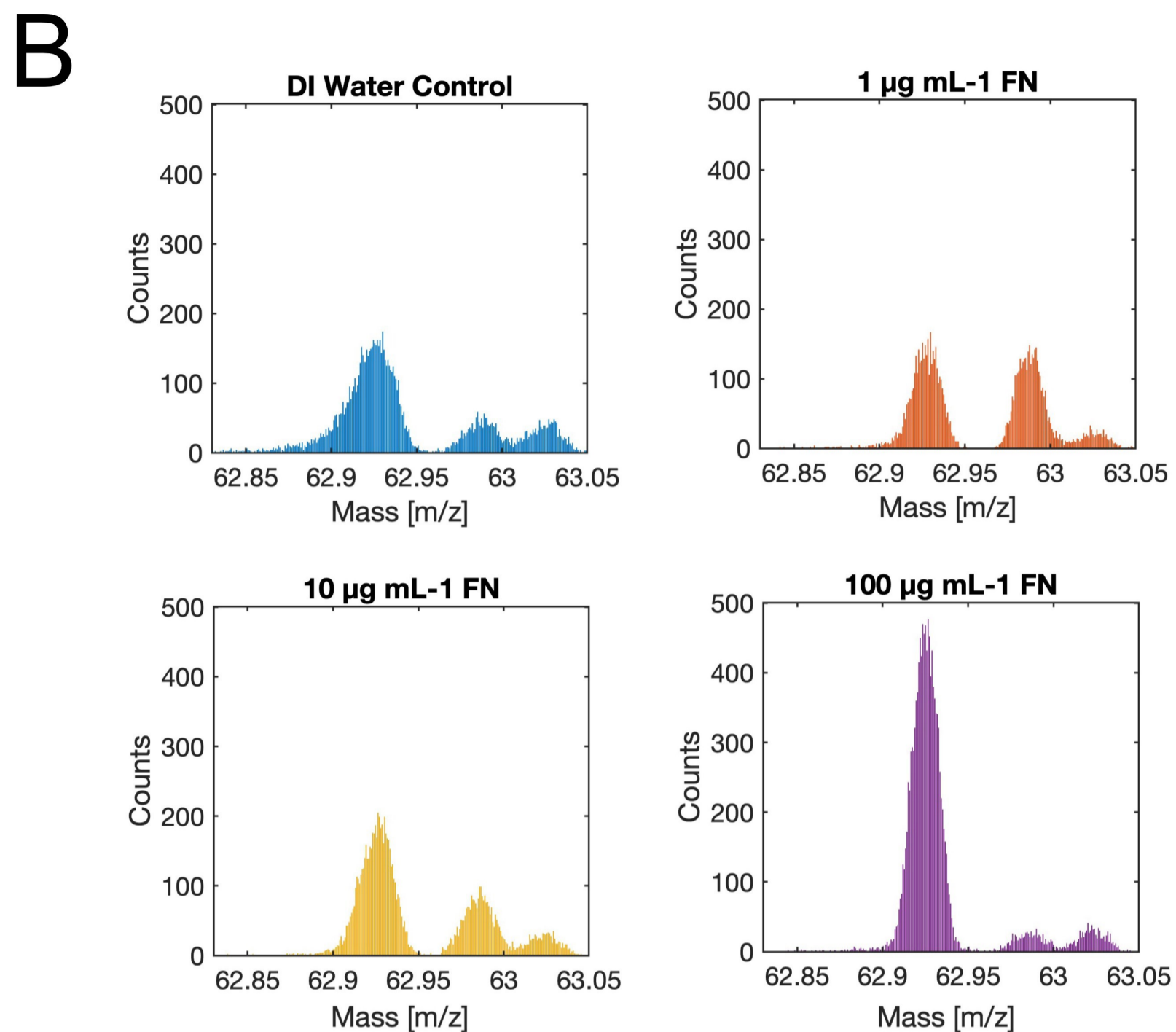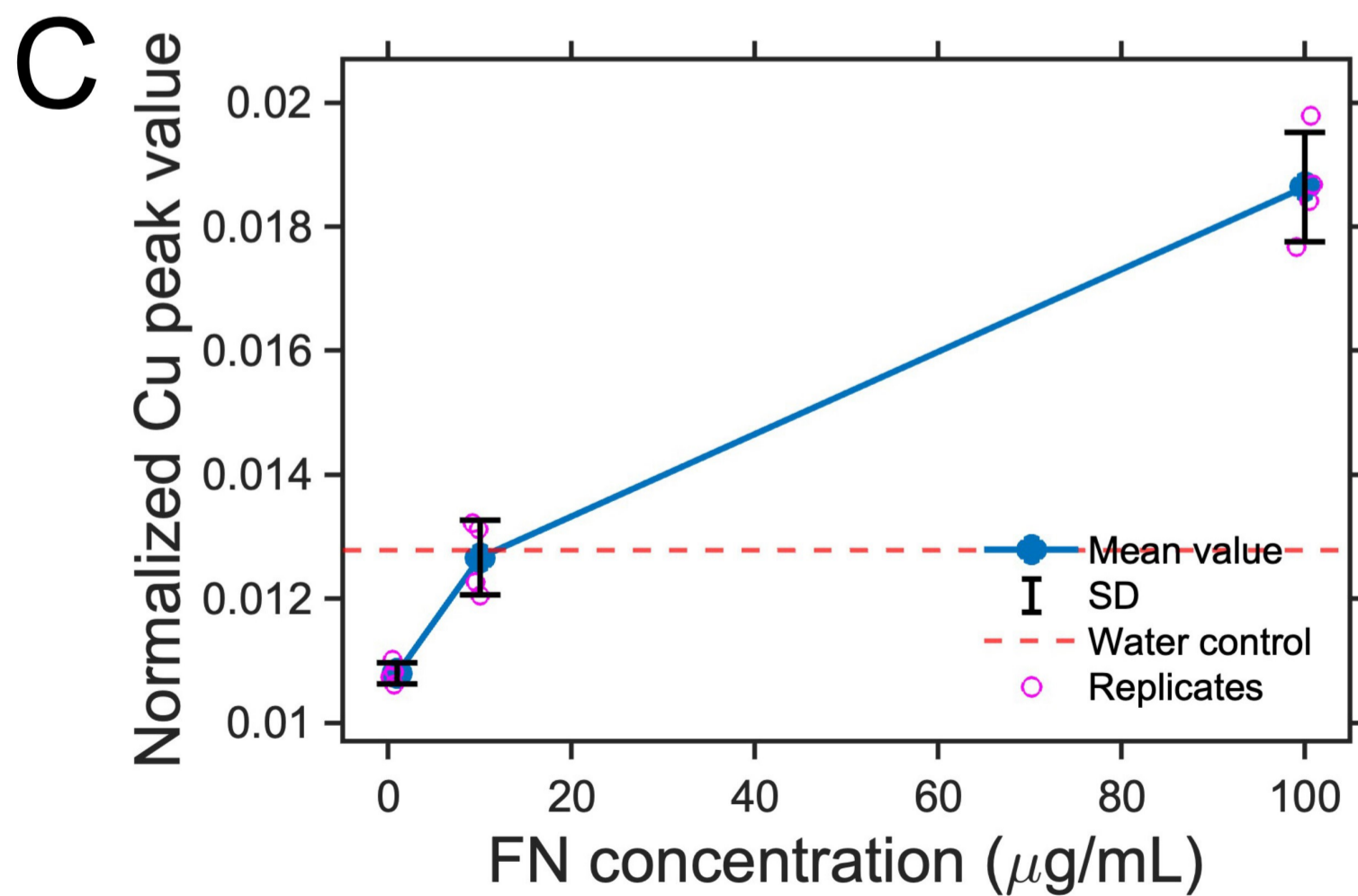

Supplement: AN-151-D5AN00962F-s003 [file AN-151-D5AN00962F-s003.pdf]

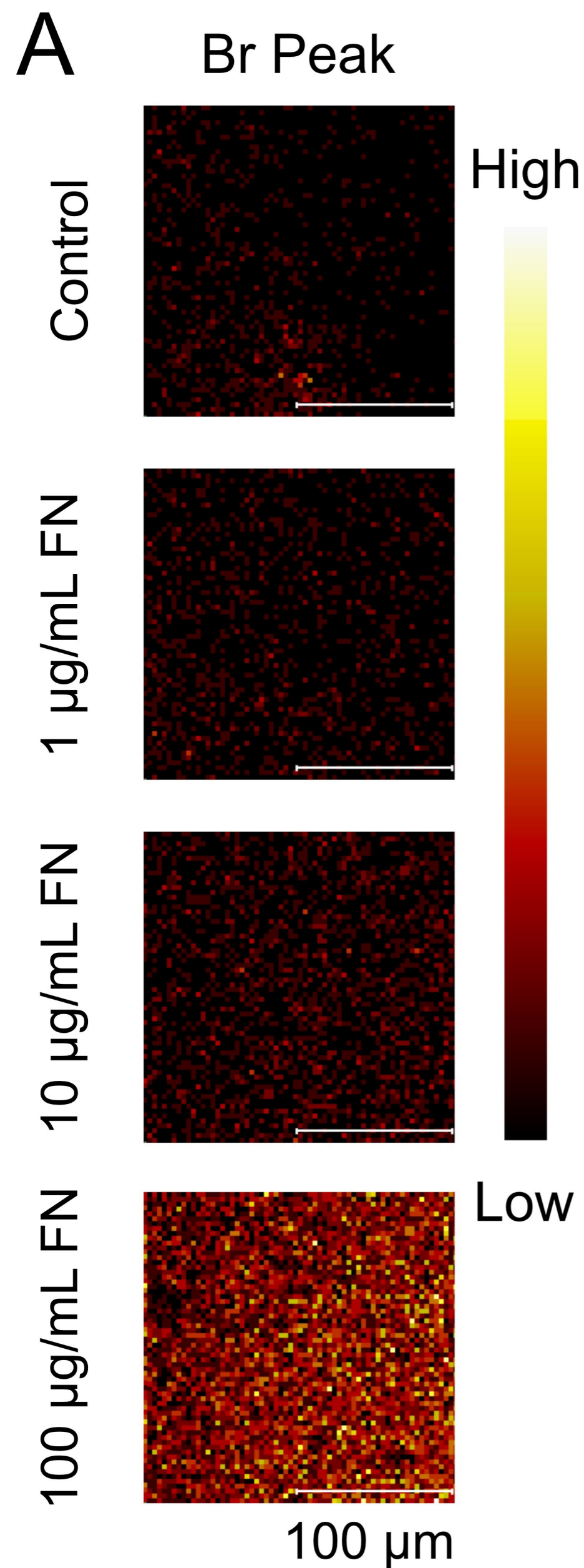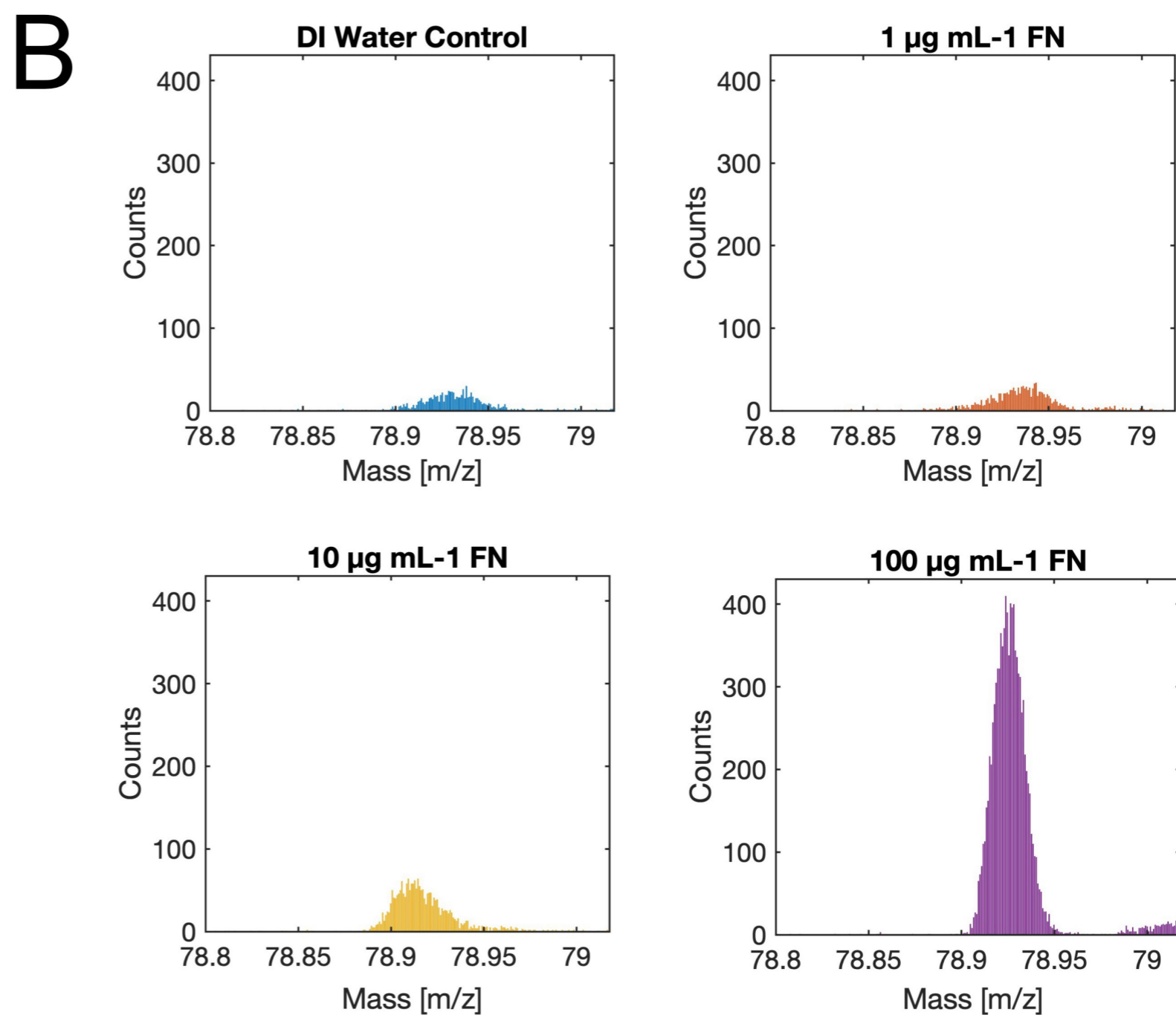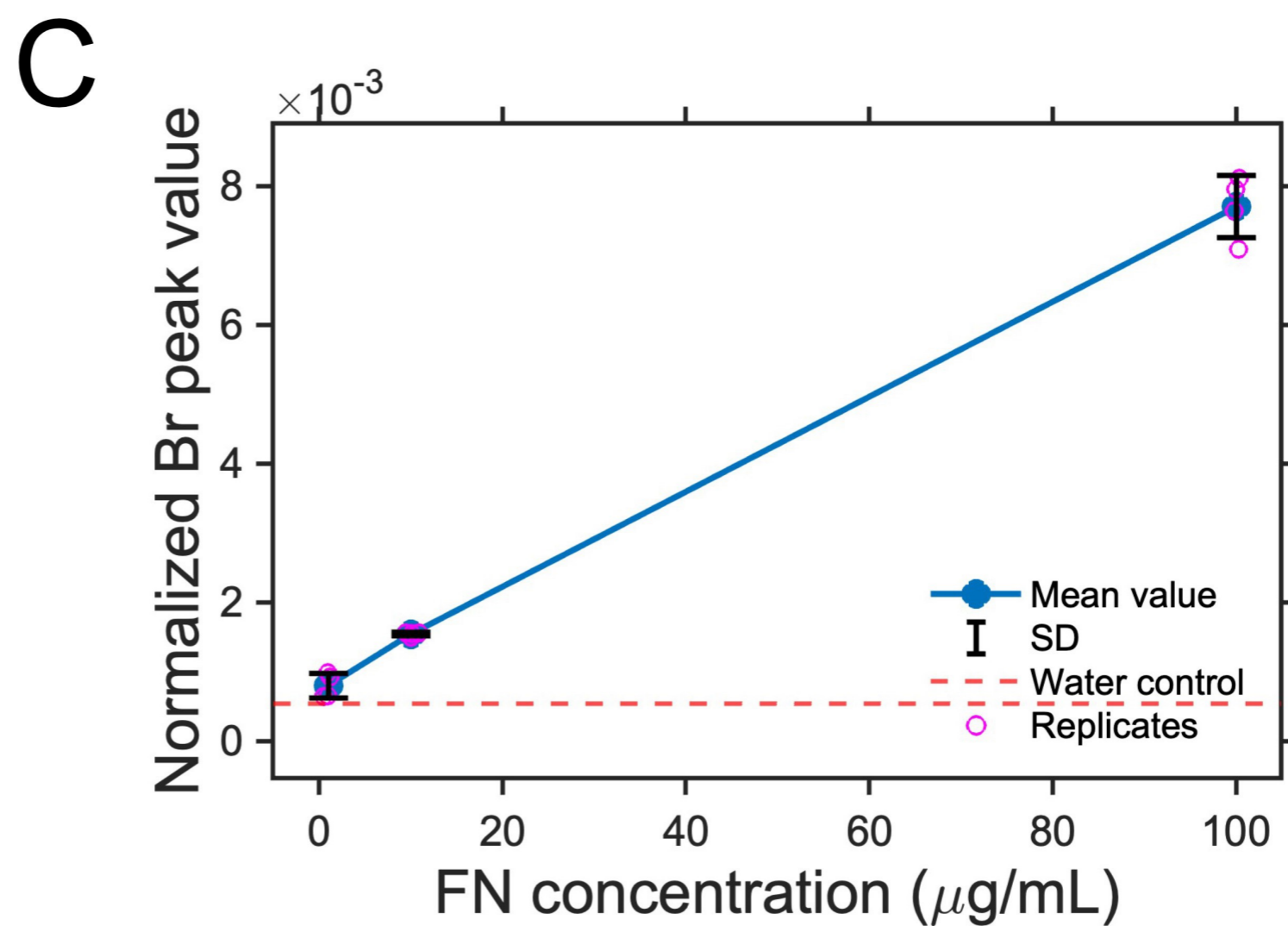

Supplement: AN-151-D5AN00962F-s004 [file AN-151-D5AN00962F-s004.pdf]

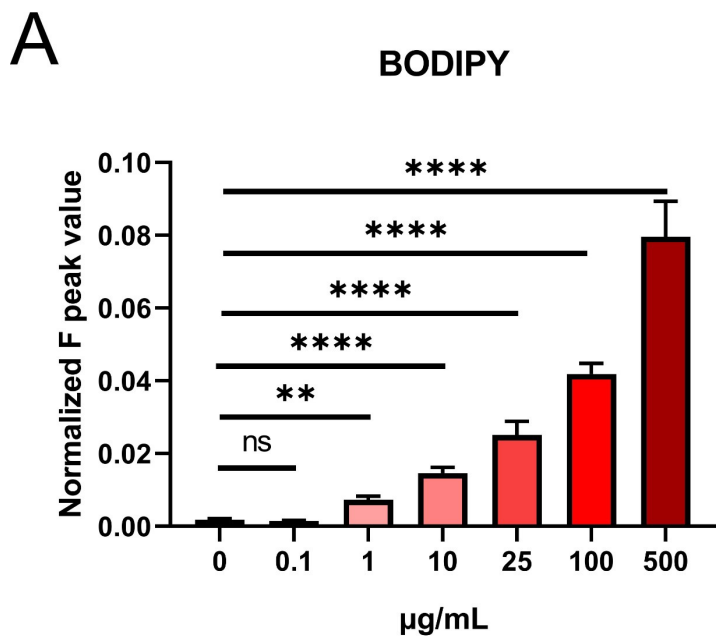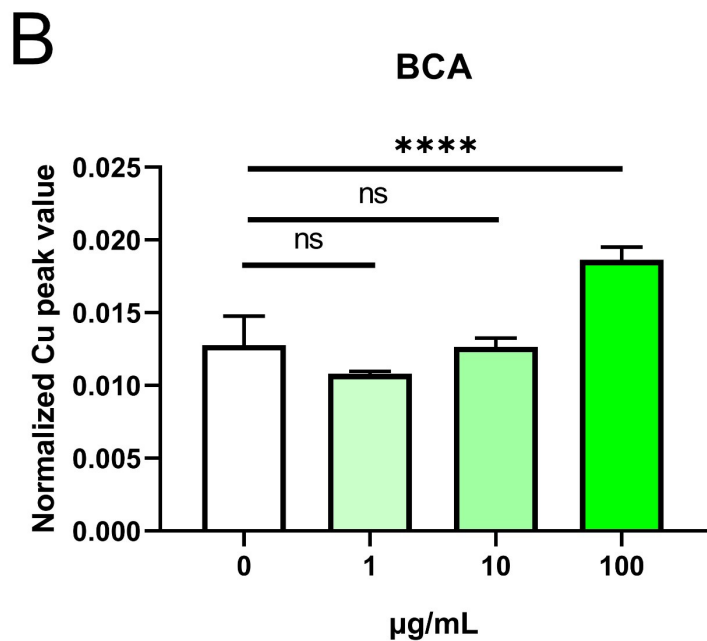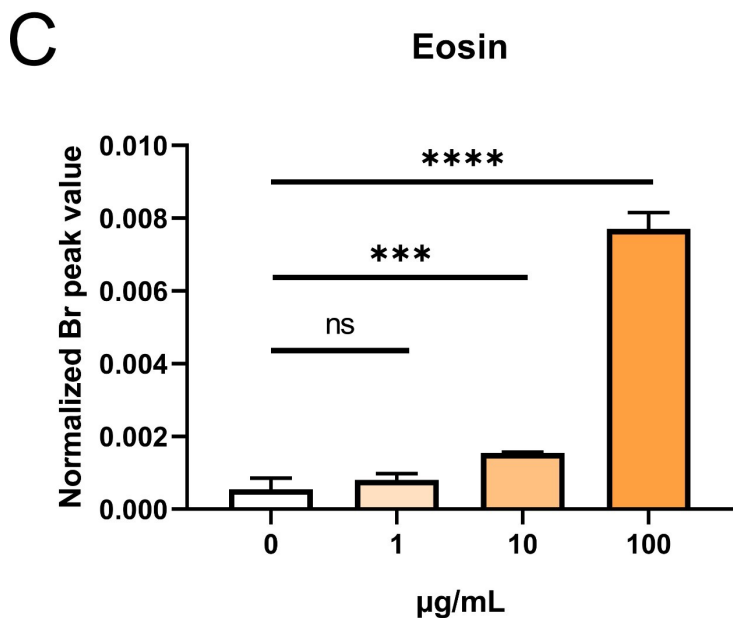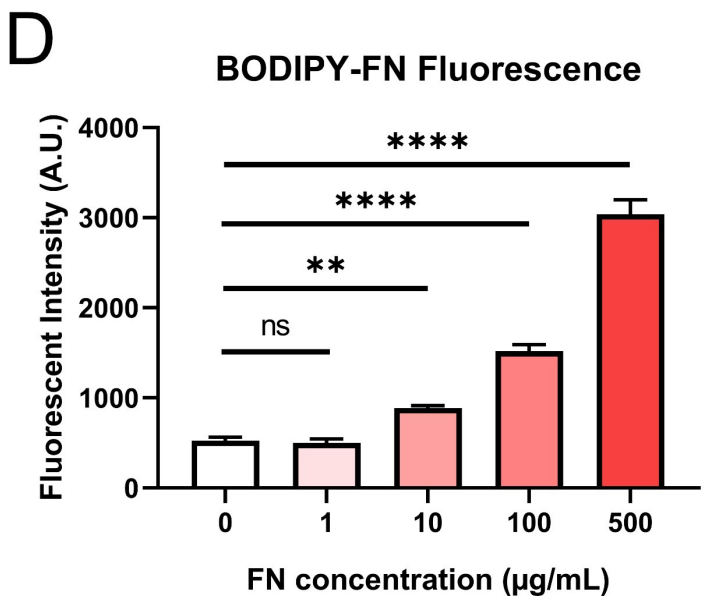

Supplement: AN-151-D5AN00962F-s005 [file AN-151-D5AN00962F-s005.pdf]

A

Data range  
0.1-500  $\mu\text{g/mL}$

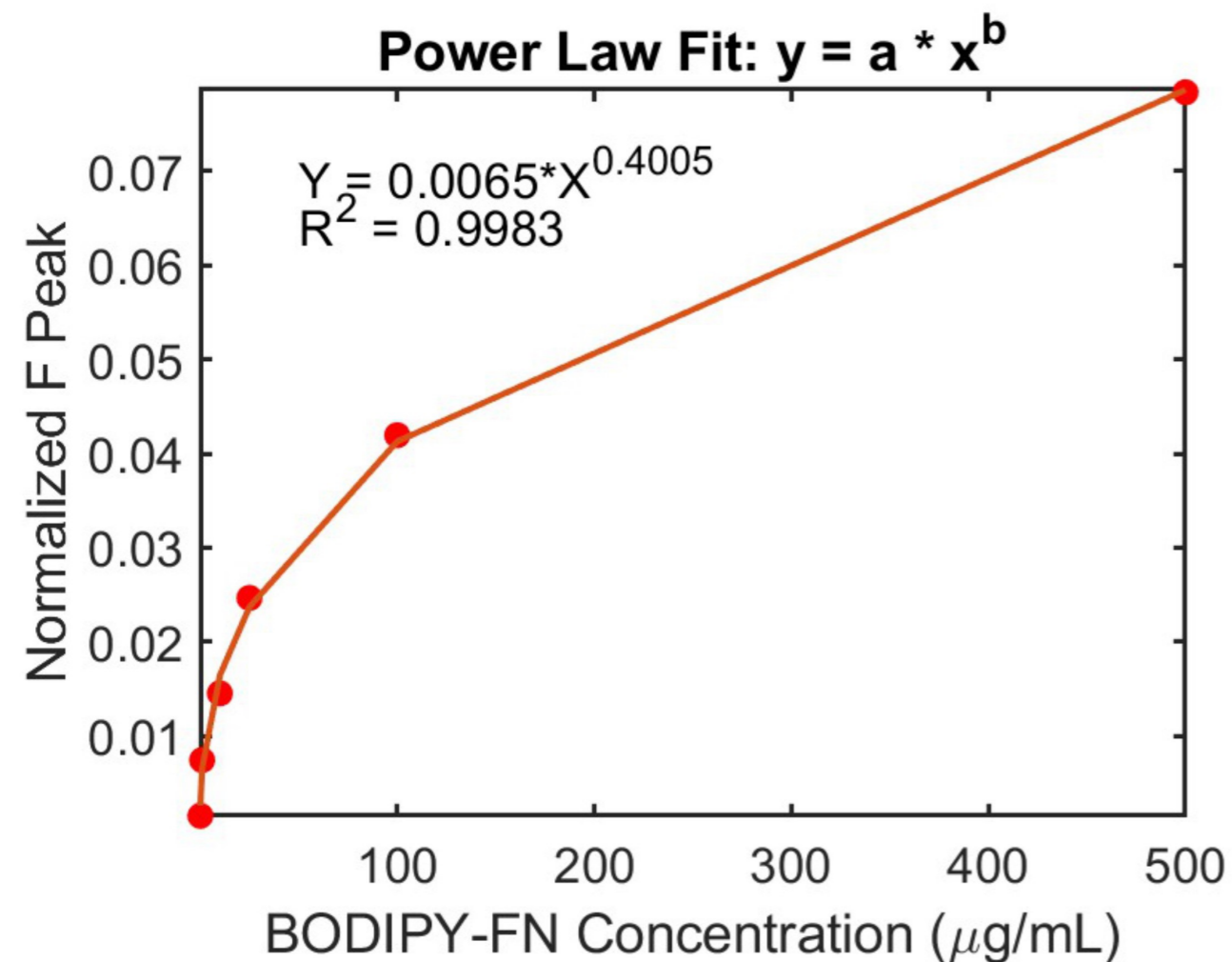

B

Data range  
0.1-25  $\mu\text{g/mL}$

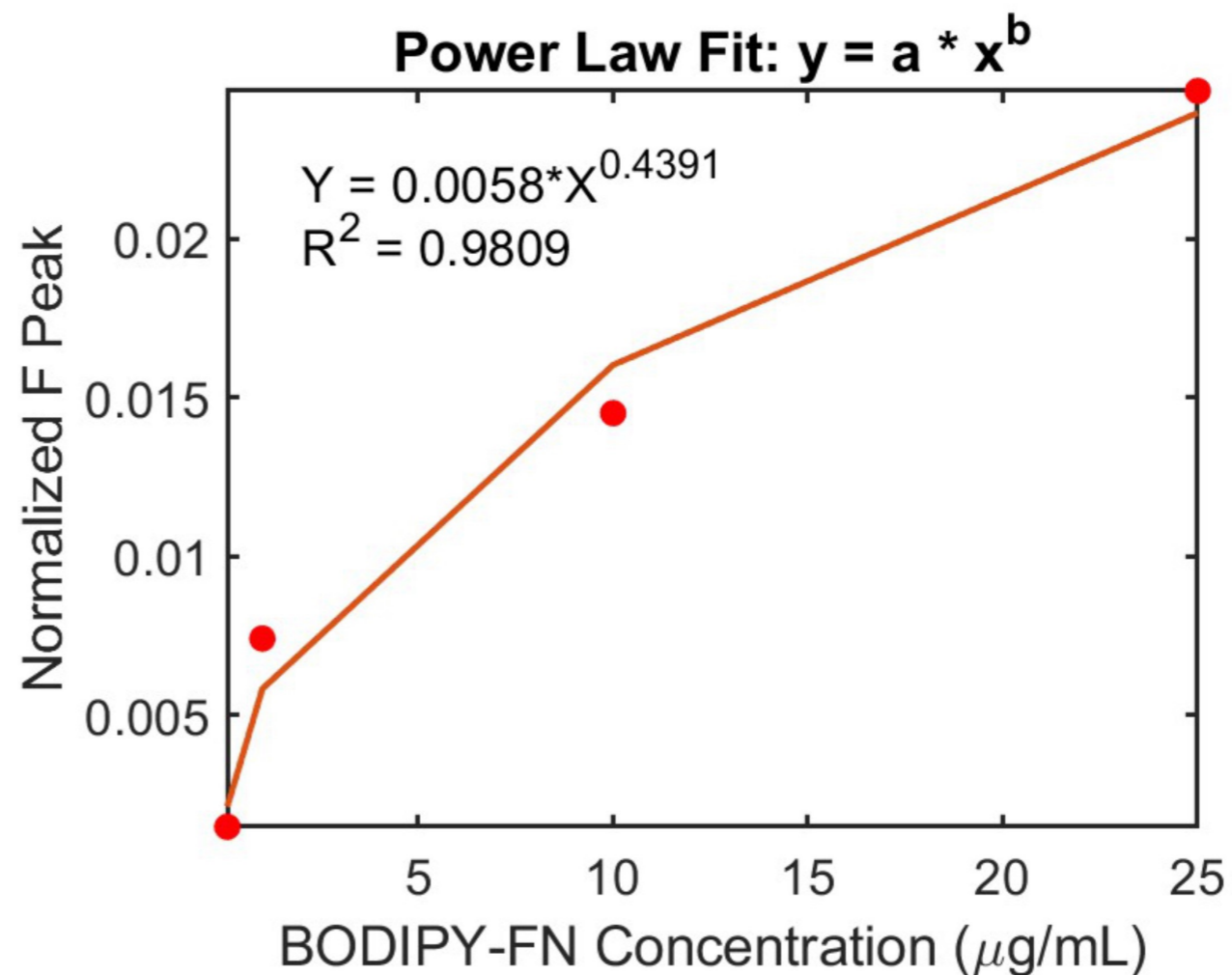

Supplement: AN-151-D5AN00962F-s006 [file AN-151-D5AN00962F-s006.pdf]

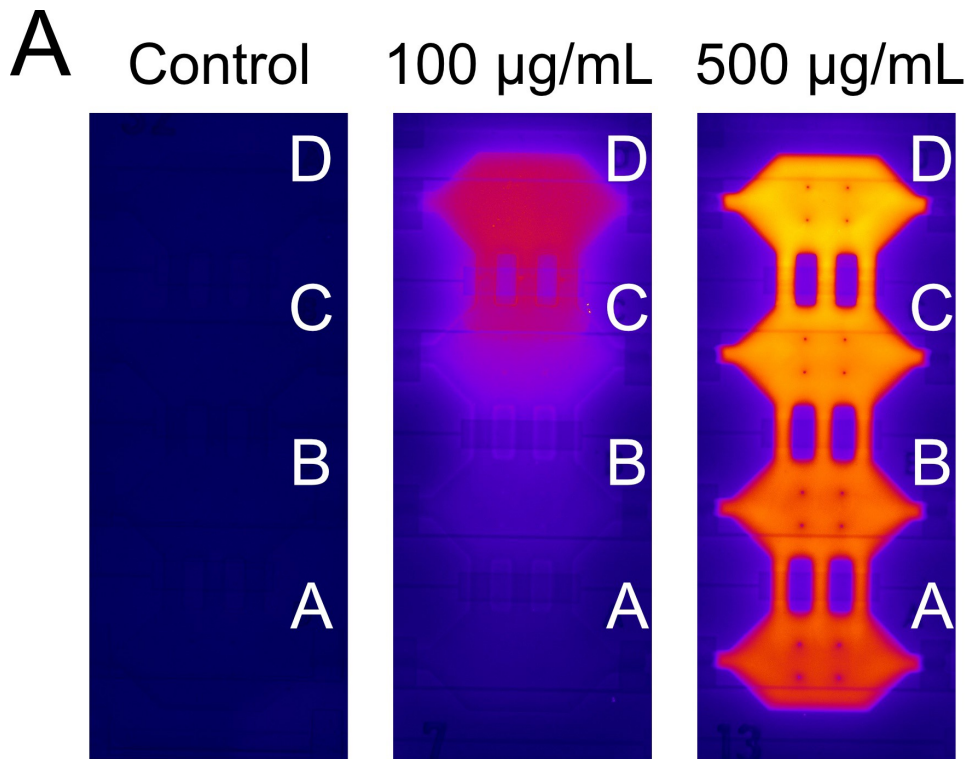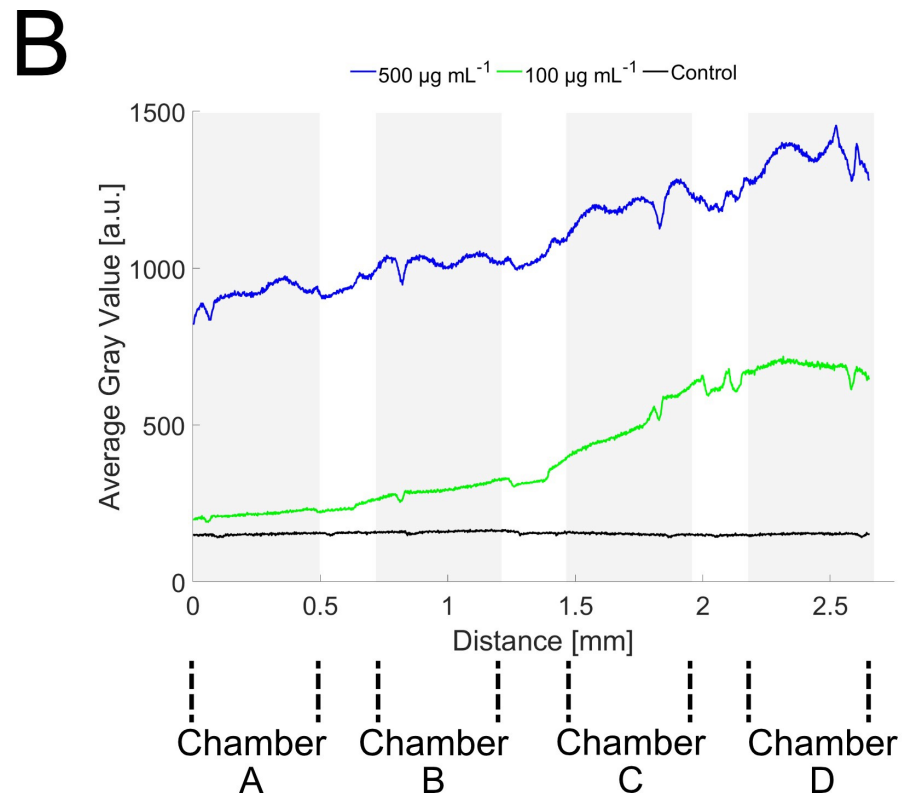

Supplement: AN-151-D5AN00962F-s007 [file AN-151-D5AN00962F-s007.pdf]

A

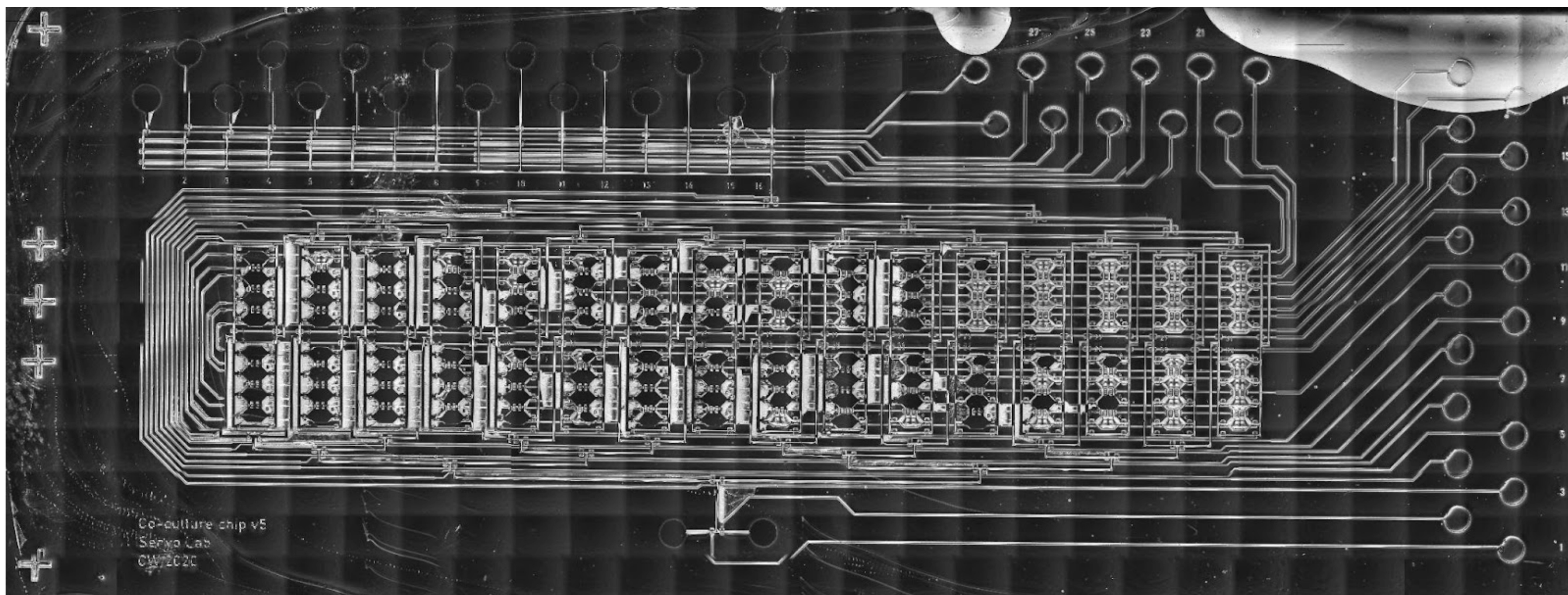

B

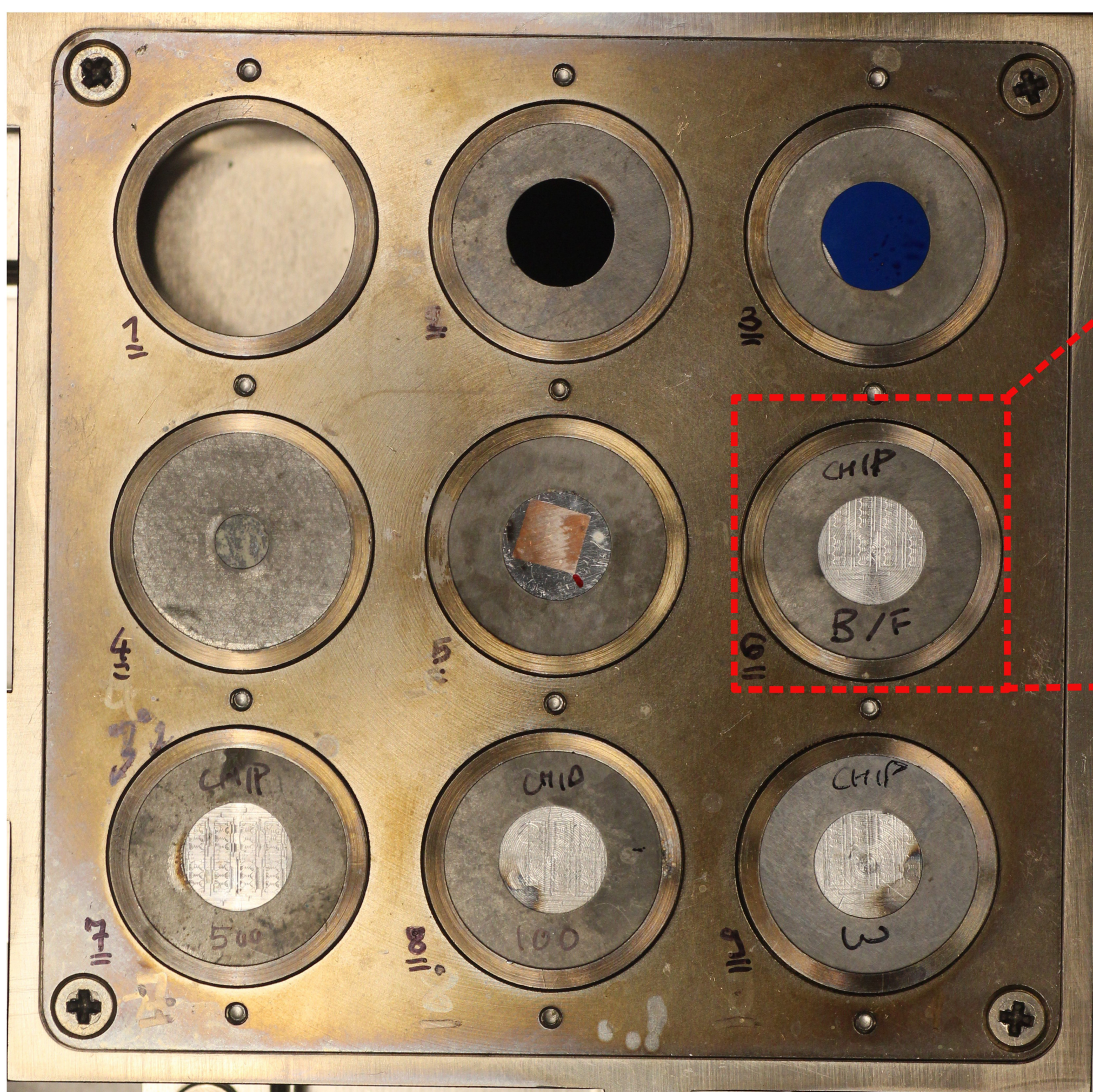

C

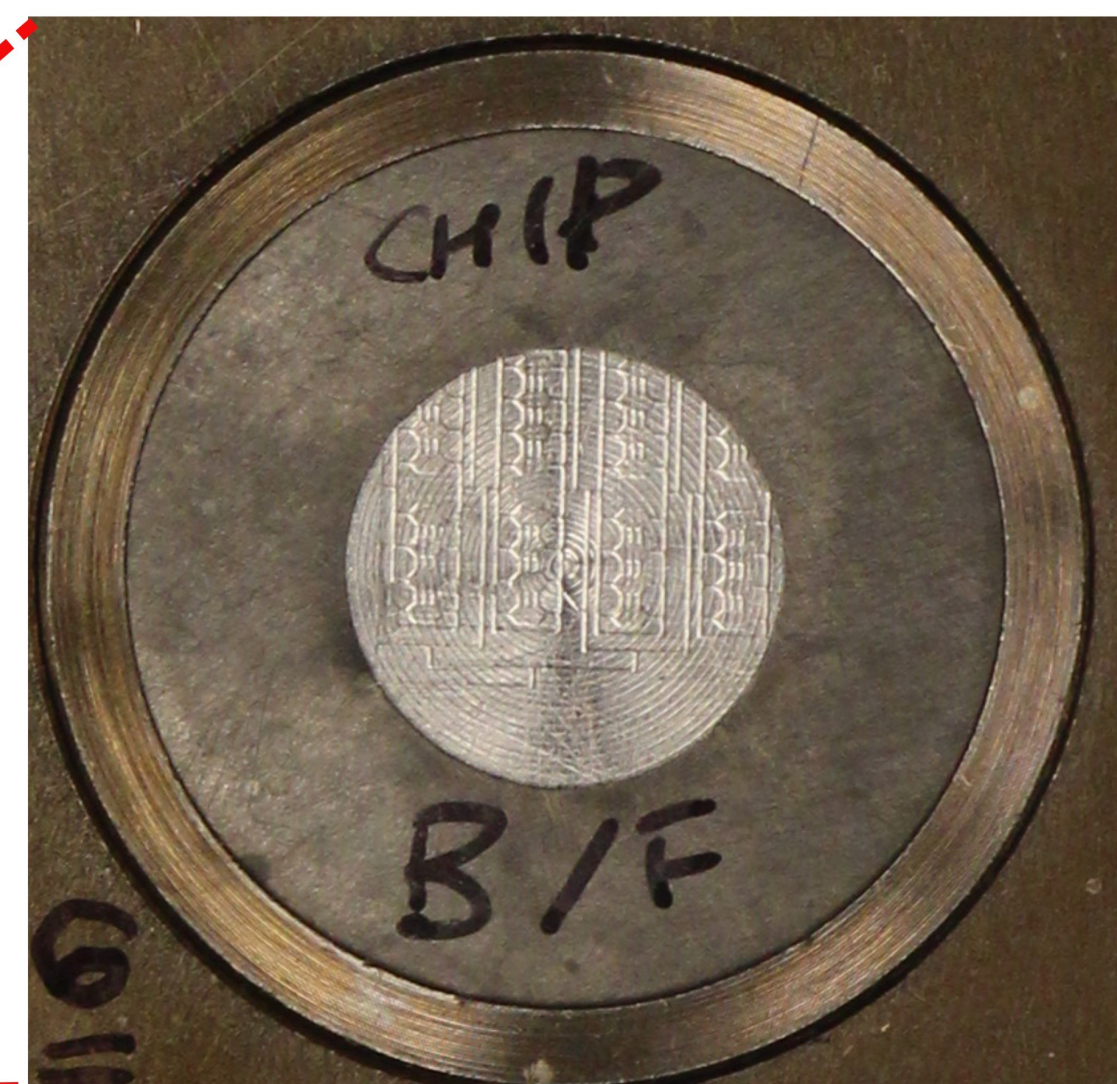

Supplement: AN-151-D5AN00962F-s008 [file AN-151-D5AN00962F-s008.pdf]
